# Plant growth promotion under phosphate deficiency and improved phosphate acquisition by new fungal strain, *Penicillium olsonii* TLL1

**DOI:** 10.3389/fmicb.2023.1285574

**Published:** 2023-10-19

**Authors:** Erinjery Jose Suraby, Valiya Nadakkakath Agisha, Savitha Dhandapani, Yee Hwui Sng, Shi Hui Lim, Naweed I. Naqvi, Rajani Sarojam, Zhongchao Yin, Bong Soo Park

**Affiliations:** ^1^Temasek Life Sciences Laboratory, National University of Singapore, Singapore, Singapore; ^2^Department of Biological Sciences, National University of Singapore, Singapore, Singapore

**Keywords:** biofertilizer, *Penicillium olsonii* TLL1, phosphate deficiency stress, phosphate solubilizing fungus, plant growth promotion

## Abstract

Microbiomes in soil ecosystems play a significant role in solubilizing insoluble inorganic and organic phosphate sources with low availability and mobility in the soil. They transfer the phosphate ion to plants, thereby promoting plant growth. In this study, we isolated an unidentified fungal strain, POT1 (*Penicillium olsonii* TLL1) from indoor dust samples, and confirmed its ability to promote root growth, especially under phosphate deficiency, as well as solubilizing activity for insoluble phosphates such as AlPO_4_, FePO_4_·4H_2_O, Ca_3_(PO_4_)_2_, and hydroxyapatite. Indeed, in vermiculite containing low and insoluble phosphate, the shoot fresh weight of Arabidopsis and leafy vegetables increased by 2-fold and 3-fold, respectively, with POT1 inoculation. We also conducted tests on crops in Singapore’s local soil, which contains highly insoluble phosphate. We confirmed that with POT1, Bok Choy showed a 2-fold increase in shoot fresh weight, and Rice displayed a 2-fold increase in grain yield. Furthermore, we demonstrated that plant growth promotion and phosphate solubilizing activity of POT1 were more effective than those of four different *Penicillium* strains such as *Penicillium bilaiae*, *Penicillium chrysogenum*, *Penicillium janthinellum*, and *Penicillium simplicissimum* under phosphate-limiting conditions. Our findings uncover a new fungal strain, provide a better understanding of symbiotic plant-fungal interactions, and suggest the potential use of POT1 as a biofertilizer to improve phosphate uptake and use efficiency in phosphate-limiting conditions.

## 1. Introduction

The availability of macro- and micronutrients plays a pivotal role in the growth and development of plants. Among the macronutrients, phosphorus is an essential element involved in metabolic processes and signal transduction pathways in plants (Nesme et al., 2018). Phosphate limitation is a major nutritional constraint as it affects plant growth development and compromises optimal yields. Phosphate deficiency in agriculture has often been addressed by applying phosphate fertilizers, but the phosphate use efficiency in plants is only 5%–25% due to the rapid fixation into insoluble forms (Schnug and Haneklaus, 2016). Moreover, the over-application has led to loss of soil fertility and associated environmental hazards (Sharma et al., 2013).

Plants respond to phosphate limitation through various physiological, morphological, and biochemical strategies. One of the acclimatization responses is the root morphology change to acquire available phosphate. The plant root morphology is remodeled by inhibiting primary root elongation and enhancing the formation of lateral roots and root hairs, which increases the effective surface area (Peret et al., 2014).

In Arabidopsis, the transcription factor STOP1 (SENSITIVE TO PROTON RHIZOTOXICITY 1) regulates citrate and malate exudation by activating the expression of its downstream genes, *MATE and ALMT1* efflux transporters, respectively, which are important for Al tolerance (Sawaki et al., 2009). In addition, RAE1 functions as an E3 ligase ubiquitinating *STOP1* and controls the expression of STOP1-regulated *AtALMT1* in Al resistance and low Pi response (Zhang et al., 2019). The inhibition of primary root elongation under phosphate stress is often linked to iron accumulation in the root apical meristem (Ward et al., 2008) which is mediated by two genes in Arabidopsis, *LPR1* (Low Phosphate Root 1) encoding multicopper oxidase with ferroxidase activity and *PDR2* (Phosphate Deficiency Response 2) encoding single P-type ATPase (Ticconi et al., 2004; Svistoonoff et al., 2007). The interaction of LPR1-PDR2 results in the accumulation of iron in the root apical meristem (RAM) and elongation zone (EZ) leading to callose deposition at the cell walls of RAM and EZ. Callose deposition impairs the expression of the transcription factor, *SHR* (Short Root), which is required for root patterning (Petricka et al., 2012). This results in blocked cell-to-cell communication and reduced RAM activity (Muller et al., 2015). Phosphate-deficiency stress also promotes the accumulation of reactive oxygen species (ROS) and peroxidase in the cell walls of the root apical meristem (RAM) and elongation zone (EZ) in Arabidopsis plants. Peroxidase activity crosslinks cell wall components, which leads to cell wall stiffening, eventually reducing root cell expansion (Balzergue et al., 2017). Phosphate deprivation also leads to higher levels of nitric oxide (NO) in the roots (Wang et al., 2010; Royo et al., 2015). NO acts as a signal molecule that mediates multiple responses, including reduction of primary roots (Wu et al., 2014), development of lateral roots (Correa-Aragunde et al., 2004), growth of root hair (Lombardo et al., 2006), and reutilization of phosphate in the cell wall (Zhu et al., 2017) in phosphate deficiency in plants.

Phosphate-solubilizing microorganisms (PSM) help in phosphate assimilation from both organic and inorganic forms of phosphates in soil, making it available for plant uptake. Soluble phosphate acquisition by PSM is attributed to several mechanisms such as chelation and ion exchange processes, production of organic acids, secretion of microbial-derived enzymes such as acid phosphatases and phytases, and production of phytohormones (Bergkemper et al., 2016). Although PSM includes both fungi and bacteria, the phosphate-solubilizing activity of fungi is comparatively higher than that of bacteria (Khan et al., 2010). Mycorrhizal associations with the plant roots are the best-characterized beneficial interactions that help to mobilize soil nutrients and improve plant growth. However, endophytic fungi are the major contributors to phosphate solubilization in the Brassicaceae family as mycorrhizal associations are rare (Bonfante and Genre, 2010). Most of the phosphate-solubilizing fungi (PSF) have been reported in the genera *Penicillium* (Li et al., 2016), *Aspergillus* (Yin et al., 2015), and *Trichoderma* (Lei and Zhang, 2015) and have been utilized as microbial inoculants in a variety of crops for improved phosphate uptake, and thereby increased biomass and yield. For example, *Penicillium oxalicum* and *Aspergillus niger* increase the bioavailability of phosphate and promote maize growth in calcareous soil (Yin et al., 2015). *Penicillium guanacastense* was reported as a potent biological fertilizer as it enhances the growth of *Pinus massoniana* under phosphate-limiting conditions (Qiao et al., 2019). Though PSF from soil has been widely investigated, the phosphate solubilizing activity of airborne fungi is less explored. Recently, *Aspergillus hydei* sp. nov. was reported to be the first phosphate-solubilizing fungus reported in the air (Doilom et al., 2020). Efficient PSF is a promising eco-friendly strategy to improve phosphate uptake and promote plant growth in modern agriculture. Nevertheless, it remains unclear how symbiotic fungi control the growth condition and regulate gene expression of their host plants under phosphate limitation.

In this study, we identify a new airborne fungal strain *P. olsonii* TLL1 (POT1) and investigate gene regulation for root growth, especially under limited phosphate availability such as phosphate deficiency and insoluble phosphate conditions in the soil, testing its phosphate solubilizing capability by fungus-plant interaction. Furthermore, we show that POT1 as a biofertilizer functions in mono/dicot plants and propose that it can be applied effectively in phosphate-limiting soils in both upland and paddy environments.

## 2. Materials and methods

### 2.1. PSF strain and plant materials

PSF was isolated from indoor dust samples and monocultured on Pikovskaya agar containing 0.5% tricalcium phosphate (CaP), pH 6.8–7.2, by incubating at 25°C for 7 days.

For seedling growth, *Arabidopsis* WT (Col-0), Bok Choy (*Brassica rapa subsp*. *chinensis*), and Rice (Temasek Rice) seeds were germinated on media containing Murashige and Skoog salts, 0.25 mM MES, 10 g/L sucrose, and 0.8% agar.

### 2.2. Identification and genotyping of PSF strain

Total genomic DNA was extracted from the PSF strain using the CTAB method (Edel et al., 2001). One hundred nanograms of genomic DNA of the PSF strain were used to amplify five genetic loci such as internal transcribed spacer (*ITS*), translation elongation factor-1-α (*TEF1*α), small ribosomal subunit (*SSU*), large ribosomal subunit (*LSU*), and Mini-chromosome maintenance protein (*MCM7*) using Hi-fidelity Phusion DNA polymerase (White et al., 1990; Schmitt et al., 2009; Raja et al., 2011 2017; Stielow et al., 2015). The genotyping was performed with primers in Supplementary Table S1. The PCR reaction profile consisted of initial denaturation at 98°C for 3 min, followed by 35 cycles of 98°C for 10 s, 55°C–68°C for 30 s, and 72°C for 1 min and a final extension of 72°C for 5 min. The sequences of PCR amplicons were determined using Sanger’s Sequencing, and the contigs were assembled using the Bioedit tool and were analyzed by BLAST searches at NCBI.

A phylogenetic tree was constructed using MEGA v. 7.0 separately for each genetic locus. The maximum-likelihood (ML) algorithm and Tamura Nei model were applied, with 1,000 bootstrap replications. Reference sequences of the genes from the genus *Penicillium* were also used for alignment. The final tree was presented with a bootstrap cutoff level above 65% (Visagie et al., 2014).

### 2.3. Colony morphology and microscopy analysis

The colony morphology of POT1 was studied by culturing it on PDA and MEA plates at 28 ± 2°C for 7 days. The colonization of POT1 was analyzed under full P conditions using co-staining with Wheat Germ Agglutinin-Alexa Fluor 488 conjugate (WGA-AF488) and propidium iodide (PI) as per the protocol mentioned in Redkar et al. (2018). Confocal analysis was performed using a TCS SP8 confocal laser scanning microscope (Leica, Germany). The excitation wavelength and detection wavelength for WGA-AF488 were 488 nm and 500–540 nm, respectively. For PI staining, the excitation wavelength and detection wavelength were 561 nm and 580–630 nm, respectively.

### 2.4. Bioassay for assessment of plant growth promoting potential

*In vitro* growth test of the Arabidopsis ecotype Col-0 was assessed in modified Hoagland’s solution under full P, low P, and -P conditions with 0.50 mM, 0.01 mM, and 0 mM of KH_2_PO_4_ (pH 5.6), respectively (Millner and Kitt, 1992). Four-days-old Arabidopsis seedlings were transferred to the test media precultured with 100 μL of POT1 conidial spore suspension (10^8^ spores/mL). The root length of the seedlings was estimated after 10 days using ImageJ software.^1^ Other growth parameters such as shoot and root fresh weight were also determined.

The total phosphorus content in the shoot was measured by digesting 45 mg of the leaf or root tissue in 1 mL of 3:1 HNO_3_/HCl in a microwave oven at 240°C for 15 min, and after digestion, it was diluted 10 times with water. The phosphorus content was determined using Agilent 720 Inductively Coupled Plasma-Optical Emission Spectrometry (ICP-OES; Agilent, United States).

### 2.5. Quantitative RT-PCR

The total RNA was isolated from shoots and roots of Arabidopsis plants grown in full P and low P conditions both with and without POT1 using FavorPrep^™^ plant total RNA mini kit (Favorgen, Taiwan). Subsequently, cDNAs were synthesized from 1 μg of total RNA samples using the iScript cDNA synthesis kit (Bio-Rad, United States). Quantitative real-time RT-PCR was then performed using the CFX96 Real-Time system (Bio-Rad, United States). Each reaction had a total volume of 20 μL, comprising 10 ng of template, 10 μL of SsoAdvanced Universal SYBR Green Supermix (Bio-Rad, United States), and 0.5 μM of each primer. The amplification conditions were set as follows: initial denaturation at 95°C for 30 s, followed by 40 cycles of denaturation at 95°C for 5 s, and annealing at 60°C for 30 s. The expression of *RAE1, STOP1, ALMT1*, and *MATE1* were analyzed with primers in Supplementary Table S2. Actin was used as the reference gene, and the gene expression level was analyzed by Genex Program (Bio-Rad, United States). The relative expression levels of each gene were normalized to their expression levels under full P conditions without POT1.

### 2.6. Determination of STOP1 protein levels in plants after POT1 inoculation

Full-length CDS of the *STOP1* gene was amplified from Arabidopsis cDNA using STOP1F: CACCATGGAAACTGAAGACGATTTGTGCAA and STOP1R: GAGACTAGTATCTGAAACAGACTCACCAAC and cloned in pENTR/D vector (Invitrogen, United States) and then transferred to pBCo-DC-3HA plant expression vector by Gateway cloning (Invitrogen, United States) using LR clonase enzyme II (Invitrogen, United States) to generate 35S: STOP1-3HA construct. The construct was then introduced into the Arabidopsis ecotype Columbia-0 (Col-0) through *Agrobacterium*-mediated floral dipping (Zhang et al., 2006). To detect STOP1 accumulation in plants, transgenic Arabidopsis seedlings harboring 35S: STOP1-3HA transgenes were grown in full P and -P conditions both with and without POT1 at pH 5.6 for 10 days. The plants (100 mg) were flash-frozen in liquid nitrogen and homogenized with 500 μL IP lysis buffer containing 1X complete protease inhibitor tablets (Roche, Germany). The total protein contents were separated on a 10% SDS-PAGE and STOP1-3HA proteins were analyzed by standard immunoblot using anti-HA antibody. Protein bands on the membrane were captured using the ChemiDoc Touch Imaging system (Bio-Rad, United States).

### 2.7. Determination of callose and NO

The roots excised from Arabidopsis plants grown under full P and low P conditions for 10 days were incubated for 2 h in 150 mM K_2_HPO_4_ and 0.01% aniline blue in the dark (Schenk and Schikora, 2015). Callose depositions were analyzed with an FV3000 confocal laser scanning microscope (Olympus, Japan) using a DAPI filter (excitation 370 nm and emission 509 nm).

For NO determination, the roots were treated with 10 μM DAF-FM DA (3-amino, 4-aminomethyl-2′, 7′-difluorescein diacetate) for 15 min, and samples were washed with HEPES buffer (pH 4.0) for 10 min (Lombardo and Lamattina, 2012). The fluorescence (excitation 495 nm and emission 515 nm) was observed using an FV3000 confocal laser scanning microscope (Olympus, Japan).

### 2.8. Analysis of insoluble phosphate-solubilizing activity of POT1

POT1 was cultured on Pikovskaya agar and NBRIP containing tricalcium phosphate (CaP), with a pH of 6.8–7.2. The colony diameter (*D*) and the diameter of the circular zone of solubilized phosphorus (*d*) were determined, and the *d*/*D* ratio was calculated to find the phosphate-solubilizing rate.

For determination of soluble P concentration in broth, POT1 was cultured on Prune agar medium (40 mL/L prune juice, 1 g/L yeast extract, 2.5 g/L lactose, 2.5 g/L sucrose, 20 g/L agar, and pH 6.5) for 2 days in the dark and 3 days in the light for sporulation. The conidial spore suspension (10^8^ spores/mL) was inoculated into 100 mL of modified Pikovskaya broth containing 1 g/L of sparingly soluble inorganic phosphates such as tricalcium phosphate (pH 7.0), iron phosphate (pH 2.5), aluminum phosphate (pH 4.0), and hydroxyapatite (pH 7.0) and incubated at 25°C and 120 rpm for 14 days. Uninoculated media with different P sources were also kept as controls. The cultures were centrifuged at 5000 rpm for 15 min, and the supernatant was filtered with a 0.22 μm syringe filter. The soluble phosphorus content was determined using phosphomolybdenum spectrophotometry.

### 2.9. Analysis of organic acids secreted by POT1

Cultures of POT1, grown under full P and low P were filtered using 0.22 μm filter, and 10 μL of the samples were analyzed on a Liquid Chromatography-Q Exactive Orbitrap Mass Spectrometer system (Thermo Fisher Scientific, United States). The multi-sampler was set to a 4°C temperature, and LC-separation was carried out using an Accucore C18 column (2.1 mm × 30 mm, 2.6 μm) at 22°C under a flow rate of 300 μL/min. The mobile phases for the reversed-phase (RP)-LC were solution A, 0.2% v/v formic acid in water; and solution B, 0.2% formic acid in 100% methanol. The MS was run in the negative mode for parallel reaction monitoring (PRM) using the ions listed in Supplementary Table S3. Calibration stock solutions were prepared and stored at −80°C, and in each batch, six-point calibration curves were generated with freshly prepared dilutions.

### 2.10. Analysis of plant growth by POT1 under insoluble phosphate conditions

For the soil test, vermiculite was used as nutrient-free soil for studying plant growth under insoluble phosphate conditions. It was washed three times with deionized water to remove any soluble P and air-dried at room temperature. To test plant growth by P solubilizing activity, POT1 mycelia (0.5 g) resuspended in 50 mL of sterile distilled water was inoculated into the vermiculite for 7 days prior to the transfer of the seedlings. To test plant growth by P solubilizing activity, POT1 mycelia (0.5 g) were drenched in vermiculite for 7 days prior to the transfer of the seedlings. Seven-days-old seedlings of Arabidopsis and Bok Choy were transferred to vermiculite and subjected to different phosphate sources.

The plants were irrigated twice a week with Hoagland’s that contained full P (0.5 mM KH_2_PO_4_), low P (0.01 mM KH_2_PO_4_), -P (0 mM KH_2_PO_4_), tricalcium phosphate (0.5 mM CaP), aluminum phosphate (0.5 mM AlP), iron phosphate (0.5 mM FeP), and hydroxyapatite (0.5 mM HA) and were grown at 22°C (Arabidopsis) and 25°C (Bok Choy) under 75% relative humidity. Growth index parameters such as shoot fresh weight, leaf area index, number of leaves, and shoot P content were analyzed.

The anthocyanin content in leaves of Arabidopsis plants was determined following the protocol by Laby et al. (2000). Leaves of 14 days-old plants, both those co-cultivated with or without POT1, were divided into three distinct groups (leaves 1–4, 5–8, and 9–12). The leaves were extracted with 99:1 methanol: HCl (v/v) overnight at 4°C. The anthocyanin contents were determined by acquiring OD at 530 and 657 nm and were calculated using the formula: OD530-(0.25 × OD657) × extraction volume (mL) × 1/weight of the tissue.

### 2.11. Effect of POT1 under local soil conditions

The growth test of Bok Choy and Temasek Rice was conducted in soil collected from Lim Chu Kang, Singapore (N 1° 26′ 7.3284″, E 103° 42′ 48.456″). Before conducting the tests, a soil nutrient analysis was performed to determine the available nutrients in Lim Chu Kang soil and commercial soil used for the cultivation of Bok Choy and Rice. One kilogram of commercial and Lim Chu Kang soils was analyzed for nitrogen, phosphorus, potassium, calcium, magnesium, sodium, copper, manganese, zinc, and boron using Mehlich 3 extraction (Mehlich, 1984) followed by Agilent 720 Inductively Coupled Plasma-Optical Emission Spectrometry (ICP-OES; Agilent, United States) analysis. Ten grams of soil was mixed with 20 mL of sterile distilled water on a magnetic stirrer for 1 h, and the pH of the soil mixture was recorded using a pH meter.

For the growth test, POT1 mycelia (0.5 g per pot) and heat-killed POT1 were incorporated into Lim Chu Kang soil a week before transferring the Bok Choy and Rice seedlings. These plants were grown under greenhouse conditions, and the corresponding growth index parameters were subsequently examined.

### 2.12. Comparison of POT1 with other *Penicillium* species

The insoluble phosphate solubilizing activity of POT1 was analyzed with other *Penicillium* strains such as *P. bilaiae* (ATCC 20851), *P. chrysogenum* (NBRC 4626), *P. janthinellum* (NBRC 31133), and *P. simplicissimum* (NBRC 106922) in CaP-amended modified Pikovskaya broth. The phosphate solubilizing rate was determined using the following formula [(soluble phosphorus concentration of treatment-soluble phosphorus concentration of control)/inorganic phosphorus concentration of each experimental group × 100] described by Qiao et al. (2019). *An in vitro* growth test was carried out to compare the effect of POT1 with other *Penicillium* strains under -P conditions.

Additionally, vermiculite soil experiments were undertaken to further assess the ability of POT1 to solubilize CaP in comparison with other strains of *Penicillium*. Vermiculite was conditioned with 0.5 g of fungal mycelia for 7 days before transplanting Arabidopsis seedlings. The plants were then watered bi-weekly with a Hoagland’s solution in which tricalcium phosphate served as the exclusive phosphorus source. Cultivated at a temperature of 22°C, growth metrics were analyzed to evaluate the effectiveness of the different strains in phosphate solubilization.

### 2.13. Statistical analysis

Statistical analysis was performed using GraphPad Prism software version 9.5 for Windows (GraphPad Software, United States). The data from replicate observations were analyzed using student’s *t*-test, and significant differences among treatments were determined at *p* < 0.05 based on an unpaired two-tailed *t*-test.

## 3. Results

### 3.1. Identification and genotyping of POT1

**Figure 1:**
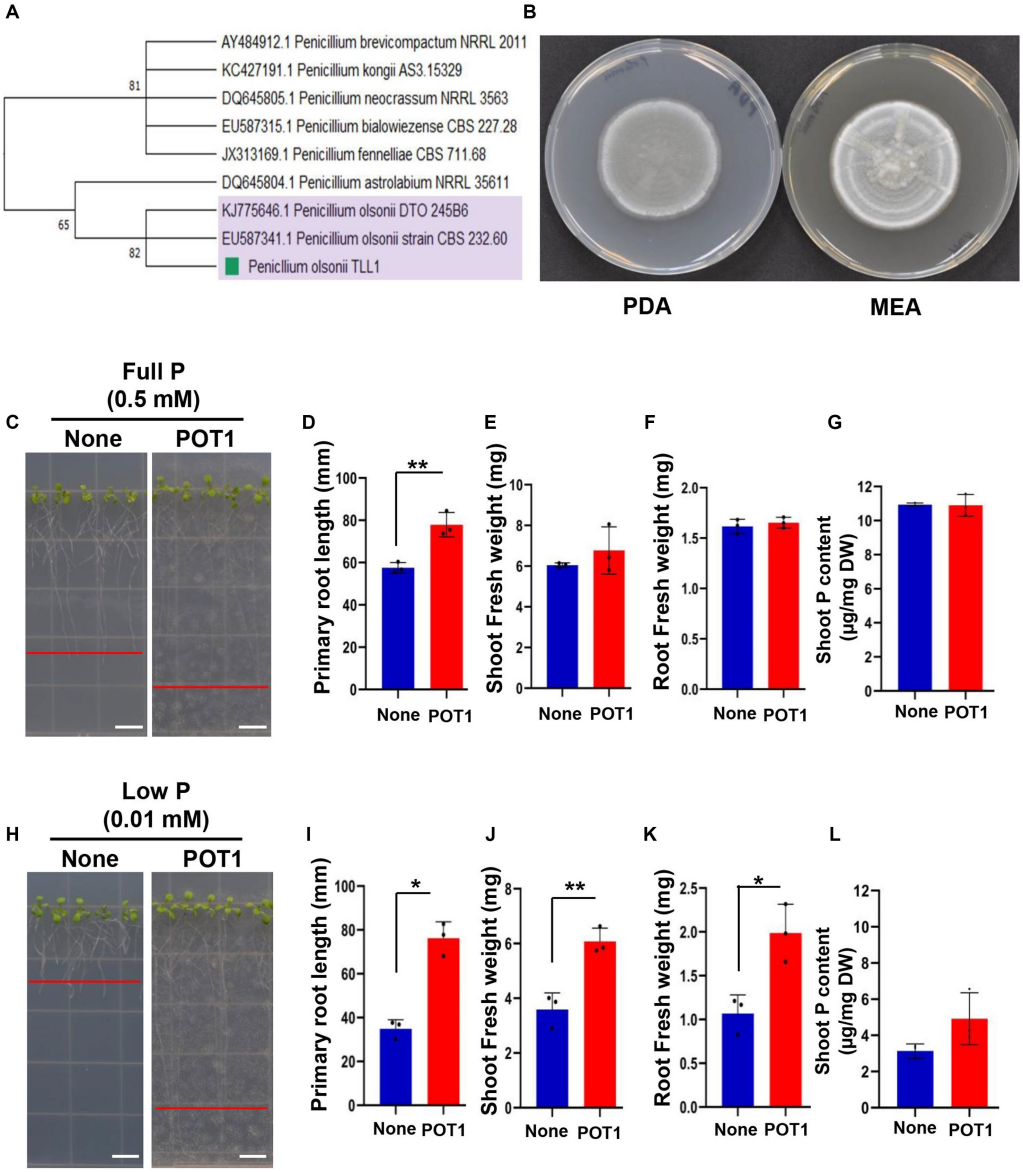
Identification of the phosphate-solubilizing fungus *Penicillium olsonii* TLL1 (POT1) and growth promotion of Arabidopsis by POT1 under P-sufficient and P-limiting conditions. **(A)** Phylogenetic tree generated from maximum likelihood (ML) analysis of POT1 based on *ITS* sequence data. Bootstrap values on 1,000 replications are shown at the nodes of the tree. **(B)** Colony morphology of POT1 cultured on (left) PDA and (right) MEA for 7 days. The scale bar represents 1 cm. **(C)** Arabidopsis plants grown in full P conditions both with and without POT1. Four-days-old plants were transferred to full P media pre-cultured with and without POT1 under *in vitro* conditions for 10 days. The scale bar represents 1 cm. The bar diagrams show **(D)** primary root length, **(E)** shoot fresh weight, and **(F)** root fresh weight of Arabidopsis Col-0 plants co-cultivated with POT1 in full P conditions for 10 days. The combined data from three independent experiments (*n* = 3; unpaired two-tailed *t*-test, ^***^p < 0.001; ^**^p < 0.01; ^*^p < 0.05) is shown. **(G)** The phosphate content in the shoot of Arabidopsis, following a 4 weeks co-cultivation with or without POT1. **(H)** Arabidopsis plants grown in low P conditions both with and without POT1. Four-days-old plants were transferred to low P media that had been either pre-cultured with POT1 or not and maintained *in vitro* for 10 days. The scale bar represents 1 cm. The bar diagrams show **(I)** primary root length, **(J)** shoot fresh weight, and **(K)** root fresh weight of Arabidopsis WT plants co-cultivated with POT1 in low P conditions for 10 days. The combined data from three independent experiments is represented (*n* = 3; unpaired two-tailed *t*-test, ^***^p < 0.001, ^**^p < 0.01, and ^*^p < 0.05). **(L)** The shoot phosphate content of Arabidopsis plants after a four-week co-cultivation with or without POT1 under low P conditions *in vitro* (*n* = 3; unpaired two-tailed *t*-test, ^**^p < 0.01).

The phylogenetic relationships between POT1 and the related fungal strains in maximum-likelihood trees were generated using five barcode markers such as *ITS*, *TEF1α*, *SSU*, *LSU*, and *MCM7*. The *ITS* sequence of POT1 clustered with two other strains of *P. olsonii* (bootstrap = 82) (Figure 1A; Supplementary Figure S1). The *TEF1α* sequences of POT1 clustered with five other strains of *P. olsonii* with a bootstrap value of 87 (Supplementary Figure S2). The *SSU* and *LSU* sequences of *P. olsonii* were also grouped with other strains of *P. olsonii* with bootstrap values of 97 and 99, respectively (Supplementary Figures S3, S4). The same or closely related *Penicillium* species were clustered as a clade on the resulting phylogenetic tree, while the out-group cluster of *Aspergillus* sp. formed non-similarity clusters. Except for *MCM7* (Supplementary Figure S5), all the sequences of the DNA barcode markers of the POT1 strain grouped with *P. olsonii* sequences, and the strain was ascertained as *P. olsonii*.

The colony morphology of POT1 was recorded after 7 days of growth on MEA and PDA (Figure 1B). When we stained the Arabidopsis roots co-cultivated with POT1 using WGA-AF488 and PI, the POT1 fungal hyphae appeared green and the plant root cell walls appeared red (Supplementary Figure S6). The co-staining revealed that POT1 colonized the surface of the roots without any penetration into the root cortical cells.

### 3.2. Plant root growth promotion by POT1 under P-sufficient and deficient conditions

We conducted experiments to assess the growth promotion of Arabidopsis mediated by *P. olsonii* TLL1 (POT1) under both P-sufficient and P-deficient media conditions (Supplementary Figure S7). Under P-sufficient conditions, Arabidopsis plants inoculated with POT1 exhibited 1.2-fold longer primary roots (Figures 1C,D), but there was no significant increase in shoot fresh weight, root fresh weight, or shoot P content compared to uninoculated plants (Figures 1E–G). Under P-deficient conditions, POT1-inoculated plants showed 2-fold longer primary root length (Figures 1H,I), and their shoot and root fresh weights significantly increased by 1.5- and 2-fold (Figures 1J,K), respectively, compared with uninoculated plants (Supplementary Table S4). Additionally, we tested the growth of Arabidopsis by POT1 inoculation under no P condition (Supplementary Figure S8A). Despite the absence of a phosphate source in the medium, the co-cultivated plants with POT1 showed 2-fold longer primary roots and 2-fold increased root fresh weight, whereas there was no difference in shoot fresh weight between inoculated and non-inoculated plants (Supplementary Figures S8B–D and Supplementary Table S5). The shoot phosphate content in plants inoculated with POT1 under no P conditions increased 1.5-fold compared to uninoculated plants (Supplementary Figure S8E).

### 3.3. Altered ALMT1 expression by POT1

**Figure 2:**
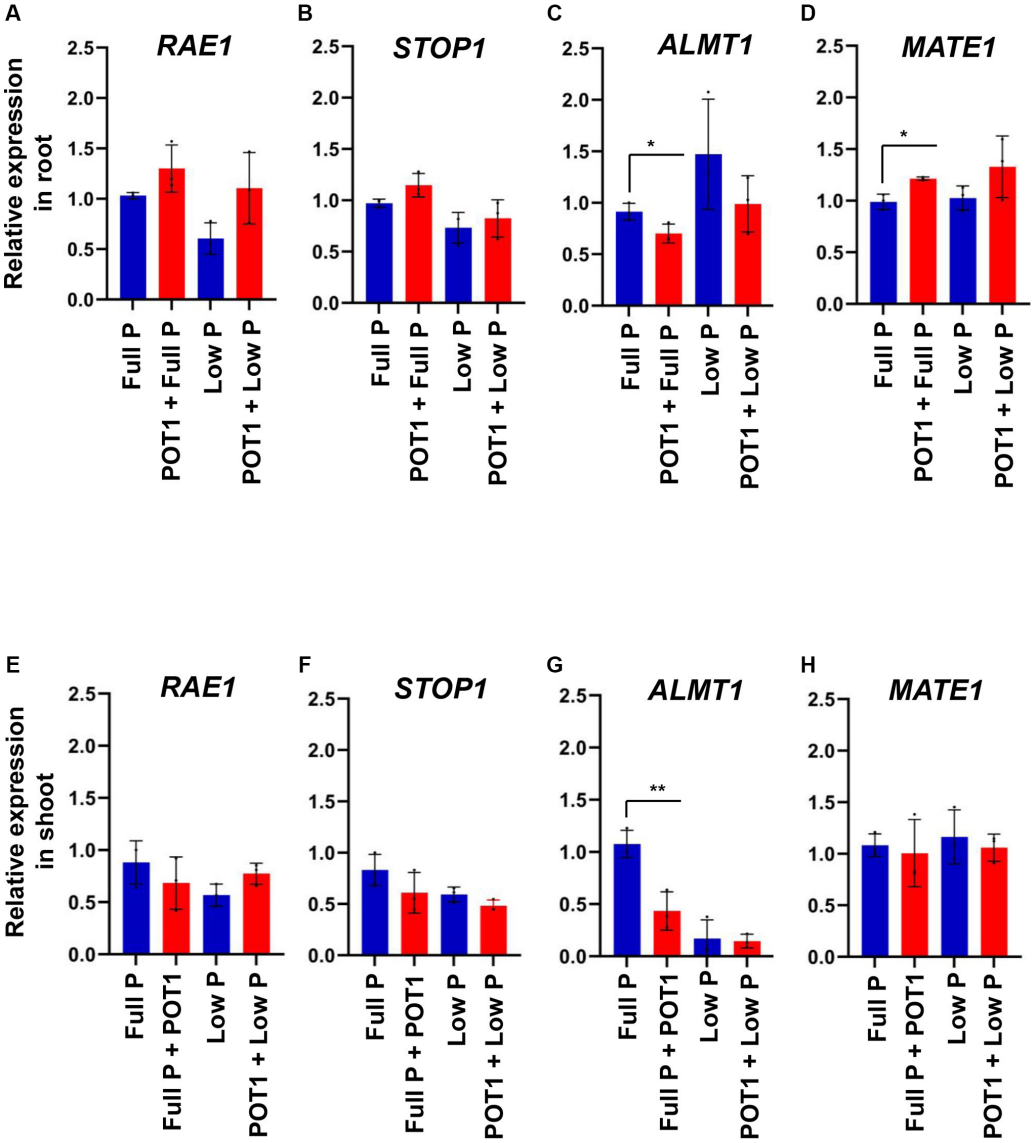
Gene expression analysis. Relative expression of **(A)** *RAE1*, **(B)** *STOP1*, **(C)** *ALMT1*, and **(D)** *MATE1* in roots of Arabidopsis when treated with and without *Penicillium olsonii* TLL1 (POT1) under full P and low P conditions. The relative expression of **(E)** *RAE1*, **(F)** *STOP1*, **(G)** *ALMT1*, and **(H)** *MATE1* in shoots of Arabidopsis when treated with POT1 under full P and low P conditions. Expression levels are shown relative to the mean expression of actin and normalized to those of full P conditions. The bar diagrams show combined data from three independent experiments (*n* = 3; unpaired two-tailed *t*-test, ^**^p < 0.01 and ^*^p < 0.05).

To identify the role of *RAE1*, *STOP1*, *ALMT1*, and *MATE1* in promoting Arabidopsis growth by POT1, we quantified their relative expression in shoots and roots using qPCR (Figure 2 and Supplementary Table S6). Our results showed that the expression level of *RAE1*, *STOP1*, and *MATE1* showed no significant changes in response to phosphate sufficiency or deficiency in either shoots or roots (Figures 2A,B,D–F,H). However, the expression of *ALMT1* was increased under phosphate-deficient conditions in the root. Consistent with the root phenotype in Arabidopsis, *ALMT1* expression was decreased upon POT1 inoculation (Figure 2C). Under the phosphate-sufficient condition, *ALMT1* expression was significantly decreased in the shoots upon POT1 inoculation (Figure 2G).

### 3.4. STOP1 protein was unstable when co-cultivated with POT1

**Figure 3:**
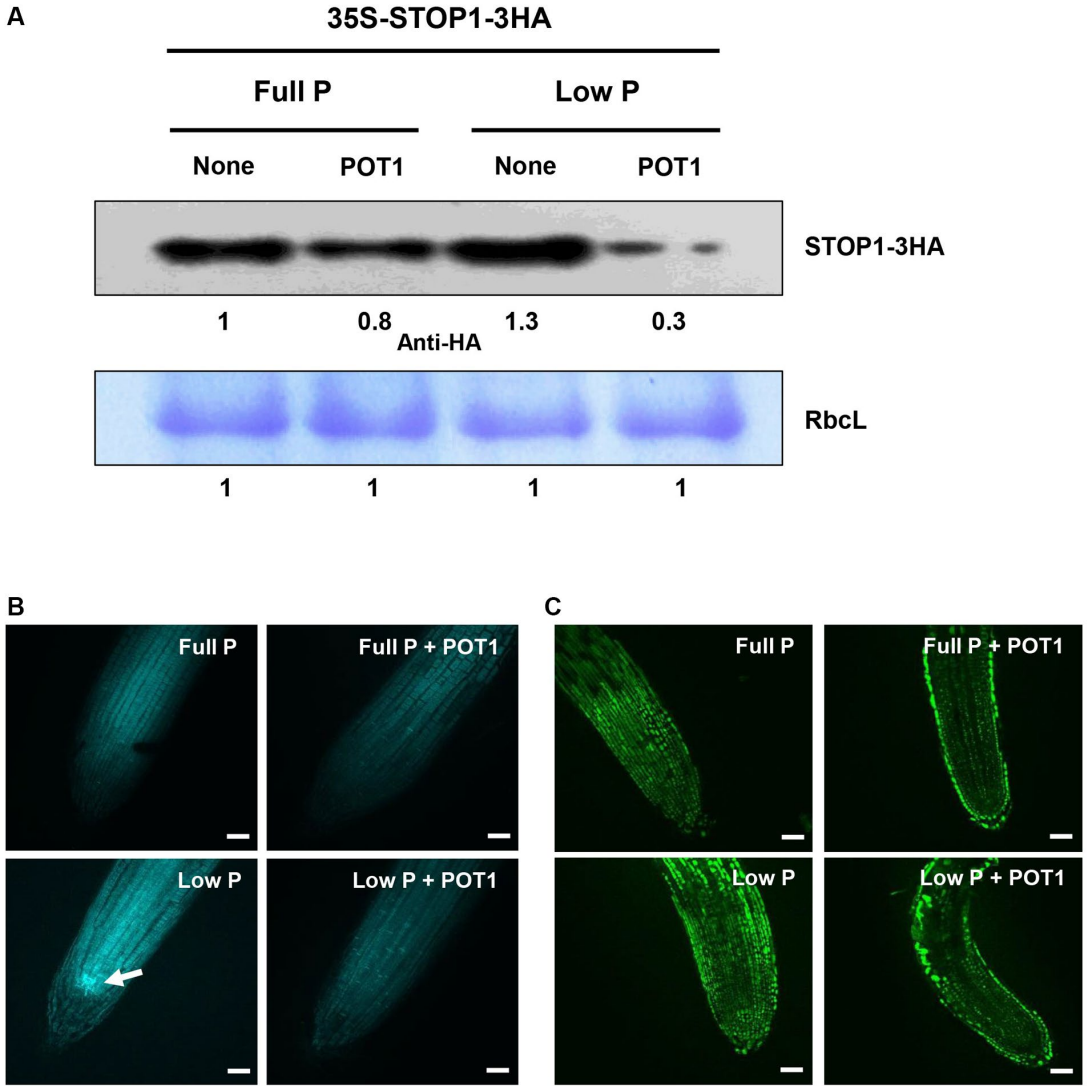
Effects of *Penicillium olsonii* TLL1 (POT1) inoculation on STOP1 accumulation, callose deposition, and NO accumulation in Arabidopsis. **(A)** Transgenic Arabidopsis plants expressing 35S: STOP1-3HA were grown under full P and low P with and without POT1 inoculation. STOP1 protein levels were analyzed by western blots using anti-HA antibodies. Stained gel bands of a large subunit of Rubisco (RbcL) were used as controls. **(B)** Callose deposition in Arabidopsis roots under full P and low P conditions, with both inoculated and uninoculated conditions of POT1. Four-days-old plants were transferred to full P media pre-cultured with and without POT1 under *in vitro* conditions for 10 days, and callose accumulation was analyzed in 1.5 cm root tips using aniline blue staining. The white arrowhead denotes callose deposition in the stem cell niche under phosphate-limiting conditions. Three independent experiments were carried out, and one representative experiment is shown. The scale bar represents 100 μm. **(C)** Nitric oxide (NO) accumulation in Arabidopsis roots under full P and low P conditions, with inoculated and uninoculated conditions of POT1. Four-days-old plants were transferred to full P media pre-cultured with and without POT1 under *in vitro* conditions for 10 days. NO accumulation was analyzed in 1.5 cm root tips using DAF-FM DA staining. The scale bar represents 100 μm.

The interaction between POT1 and STOP1 protein under full P and low P conditions was demonstrated in the transgenic Arabidopsis plant. The STOP1 protein was found to be degraded by POT1 under both phosphate-sufficient and deficient conditions (Figure 3A). Under phosphate-deficient conditions, POT1 induced a greater decrease in STOP1 protein levels compared to phosphate-sufficient conditions.

### 3.5. Inhibition of callose deposition and NO accumulation by POT1

To confirm that POT1 inhibits the negative components of root growth, we investigated its effect on callose deposition and nitric oxide (NO) accumulation under phosphate-sufficient and deficient conditions. Under phosphate deficiency, POT1-inoculated plants showed decreased callose deposition in the root apical meristem compared to the massive callose formation observed in uninoculated plants (Figure 3B). However, under phosphate-sufficient conditions, no difference in callose deposition was observed in plants with or without POT1. The capability of POT1 to inhibit endogenous NO accumulation in roots was tested using the fluorescent probe DAF-FM DA. Green fluorescence was clearly observed under phosphate-deficient conditions, indicating phosphate-dependent NO production. In contrast, after inoculating POT1, the NO level in the root decreased, suggesting the involvement of POT1 in root development under phosphate deficiency (Figure 3C).

### 3.6. Quantitative assay for phosphate-solubilizing activity of POT1

**Figure 4:**
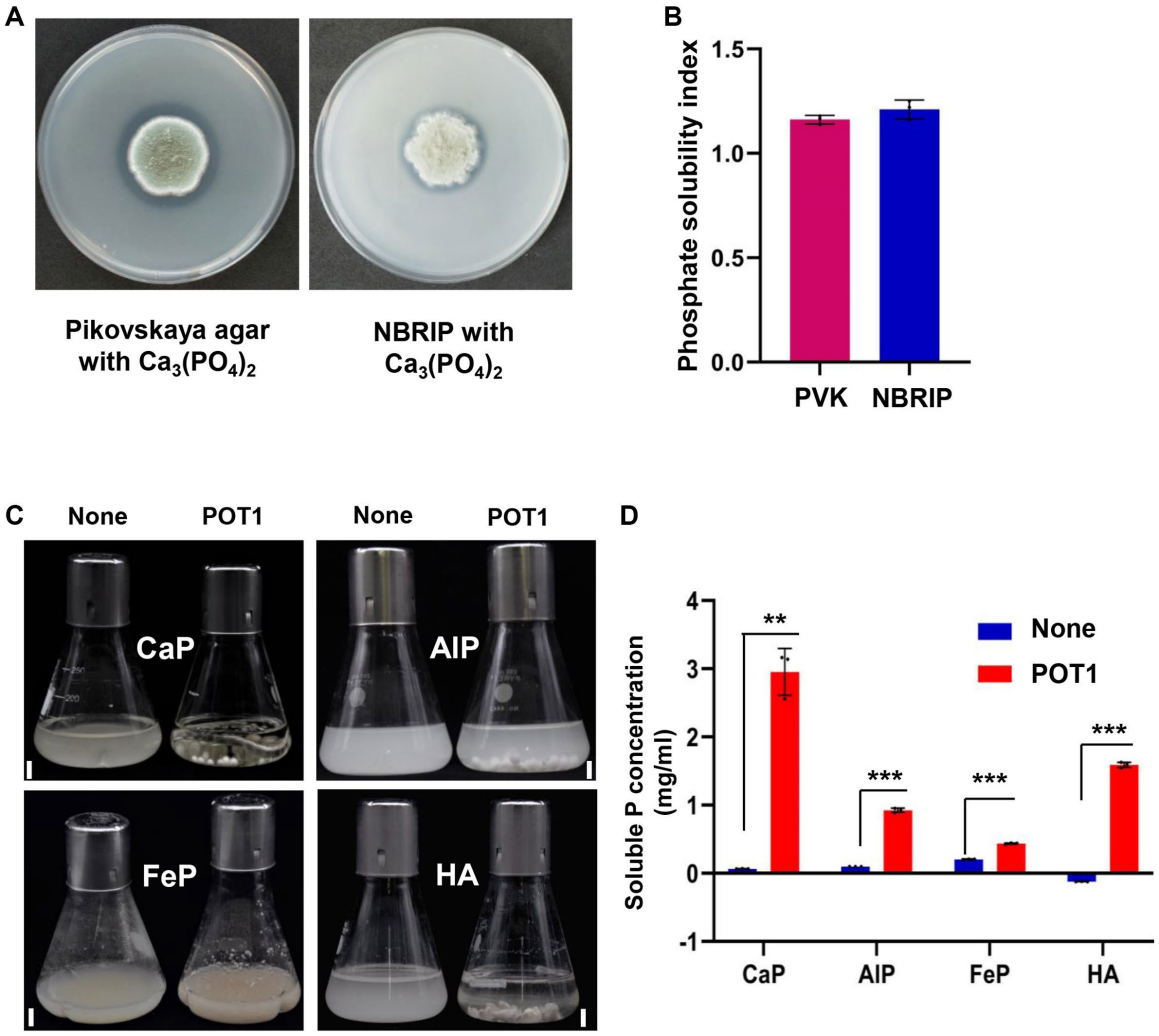
Insoluble phosphate solubilization activity of *Penicillium olsonii* TLL1 (POT1) under different recalcitrant P sources. **(A)** POT1 producing dissolved circular zones of phosphorus when cultured on Pikovskaya agar and NBRIP media amended with CaP for 7 days. **(B)** A bar diagram showing phosphate solubility indices of POT1 when cultured in Pikovskaya agar and NBRIP media amended with CaP for 7 days. **(C)** Phosphate-solubilizing potential of POT1 in various inorganic phosphorus compounds such as tricalcium phosphate (CaP), aluminum phosphate (AlP), iron phosphate (FeP), and hydroxyapatite (HA) after 14 days. Scale bar represents 1 cm. **(D)** Soluble P concentration produced by POT1 in different inorganic insoluble phosphates such as CaP, AlP, FeP, and HA. The POT1 spore suspension was inoculated in 100 mL-modified Pikovskaya media amended with CaP, AlP, FeP, and HA for 2 weeks and the soluble phosphate concentration was measured using phosphomolybdenum spectrophotometry. Three biological replicates of each treatment were analyzed (*n* = 3; unpaired two-tailed *t*-test, ^***^p < 0.001, ^**^p < 0.01, and ^*^p < 0.05). The error bars show the standard deviation.

When POT1 was grown on PVK and NBRIP media amended with Ca_3_(PO_4_)_2_ for 7 days at 25°C, visible halo zones of dissolved phosphorus were observed, indicating recalcitrant phosphate solubilization (Figure 4A). When cultured on Pikovskaya and NBRIP media, POT1 showed a phosphate solubility index (*d*/*D* ratio) of 1.161 and 1.212, respectively (Figure 4B and Supplementary Table S7).

POT1 was found to solubilize tricalcium phosphate [Ca_3_(PO_4_)_2_], hydroxyapatite [Ca_10_(PO_4_)_6_ (OH)_2_], aluminum phosphate (AlPO_4_), and iron phosphate (FePO_4_·4H_2_O) accompanied by a decline in the pH of the supernatants in modified PVK media (Supplementary Table S8). POT1 exhibited the highest phosphate-solubilizing activity in media containing Ca_3_(PO_4_)_2_, followed by hydroxyapatite, aluminum phosphate, and iron phosphate (Figure 4C). In CaP and hydroxyapatite, the soluble phosphorus concentrations were 2.95 mg/mL and 1.574 mg/mL, respectively. POT1 could also solubilize AlPO_4_ and FePO_4_, releasing soluble P at concentrations of 0.924 mg/mL and 0.436 mg/mL, respectively, after 14 DPI (Figure 4D). The phosphate-solubilizing rate was 57% for CaP, 31% for hydroxyapatite, 20% for AlP, and 3% for FeP (Supplementary Table S8).

### 3.7. Secretion of organic acids by POT1

The organic acids released by POT1 were measured under both full P and low P conditions. The secretion of gluconic acid (238 mg/L) was higher under low P conditions, whereas the secretion of citric acid (212 mg/L) was higher under full P conditions (Supplementary Figure S9). The malic acid concentration in low P (3 mg/L) was reduced 45-fold when compared to full P conditions (136 mg/L). Similarly, the amount of succinic acid secreted by POT1 into low P culture filtrate was reduced 25-fold.

### 3.8. Growth promotion in Arabidopsis and leafy vegetables by POT1 under insoluble phosphate conditions

**Figure 5:**
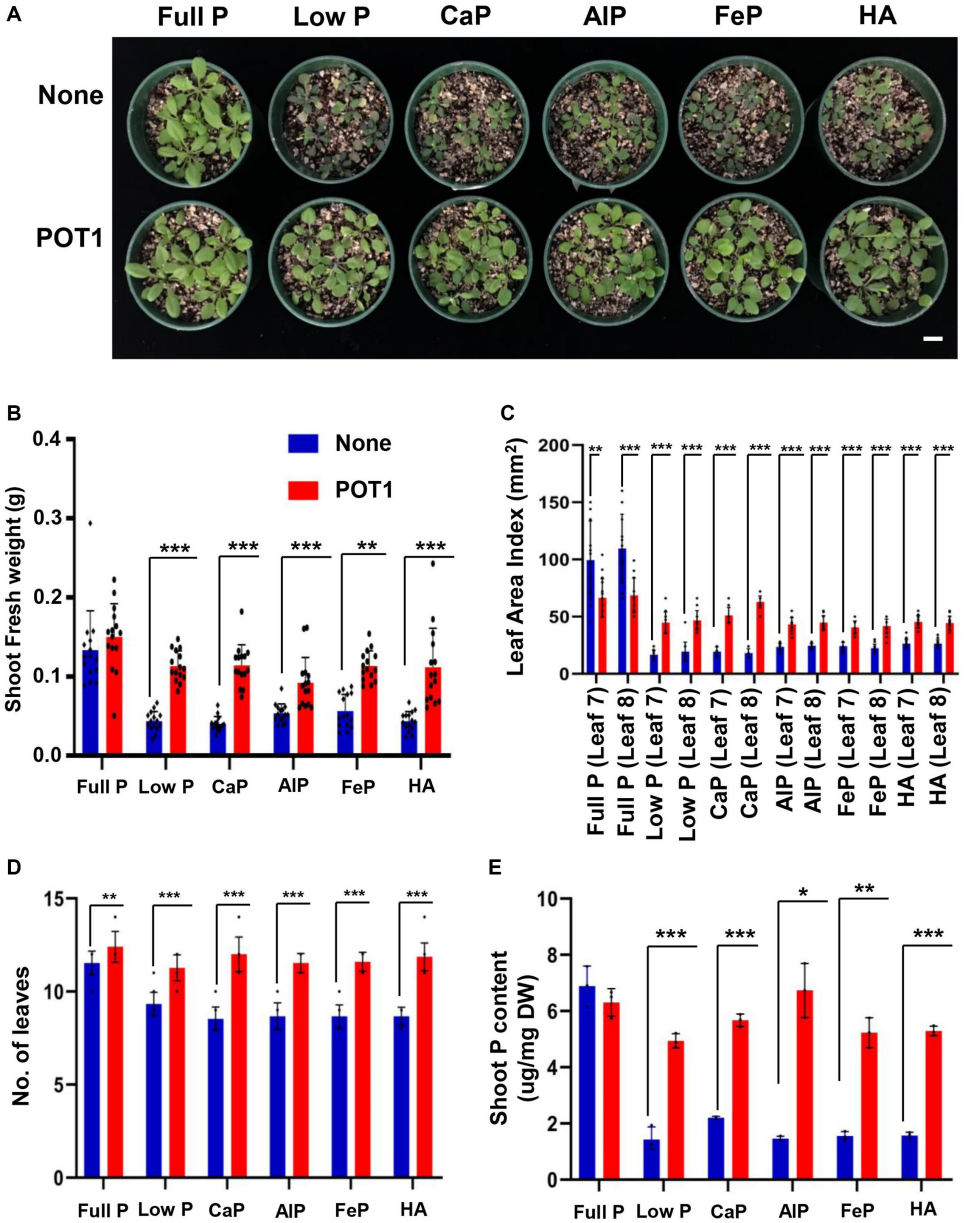
*Penicillium olsonii* TLL1 (POT1) promotes the growth of Arabidopsis under insoluble phosphate and phosphate-limiting conditions. **(A)** A representative image of *A. thaliana* WT plants grown in full P, low P, CaP, AlP, FeP, and HA as the sole source of P with and without POT1. Ten-days-old plants were transferred to vermiculite soil pre-inoculated with and without POT1 and were harvested after 2 weeks. The scale bar represents 1 cm. The bar diagrams represent **(B)** shoot fresh weight, **(C)** leaf area Index, and **(D)** number of leaves of *A. thaliana* WT plants grown in full P, low P, CaP, AlP, FeP, and HA with and without POT1 in vermiculite for 2 weeks. The combined data from 15 independent experiments were analyzed (*n* = 15; unpaired two-tailed *t*-test, ^***^p < 0.001, ^**^p < 0.01, and ^*^p < 0.05). **(E)** Shoot phosphate content of Arabidopsis plants grown in full P, low P, CaP, AlP, FeP, and HA with and without POT1 after 4 weeks (*n* = 3; unpaired two-tailed *t*-test, ^**^p < 0.01).

To investigate the growth promotion and phosphate-solubilizing capability of POT1 on Arabidopsis growth under P-sufficient and deficient conditions including insoluble P sources, we co-cultivated Arabidopsis plants in vermiculite, which is a nutrient-free soil (Figure 5 and Supplementary Table S9). Arabidopsis plants grown in low P and insoluble P sources such as tricalcium phosphate (CaP), aluminum phosphate (AlP), iron phosphate (FeP), and hydroxyapatite (HA) conditions are stunted without POT1 inoculation (Figure 5A). Consistent with Arabidopsis growth condition, shoot fresh weight, leaf index, number of leaves, and shoot phosphate content were higher in POT1 inoculated plants compared to non-inoculated plants (Figures 5B–E; Supplementary Figure S10). The anthocyanin accumulation, a typical phenotype of plants grown under phosphate deficiency (Jiang et al., 2007), was lower in POT1-inoculated plants than in non-inoculated plants (Supplementary Figure S11 and Supplementary Table S10). Under no P condition in vermiculite soil, the shoot fresh weight, the number of leaves, leaf area index, and shoot P content of POT1-inoculated Arabidopsis plants were increased significantly, compared with non-inoculated plants (Supplementary Figure S12 and Supplementary Table S11). However, plants grown under no P condition still showed growth defects when compared with phosphate-sufficient plants.

**Figure 6:**
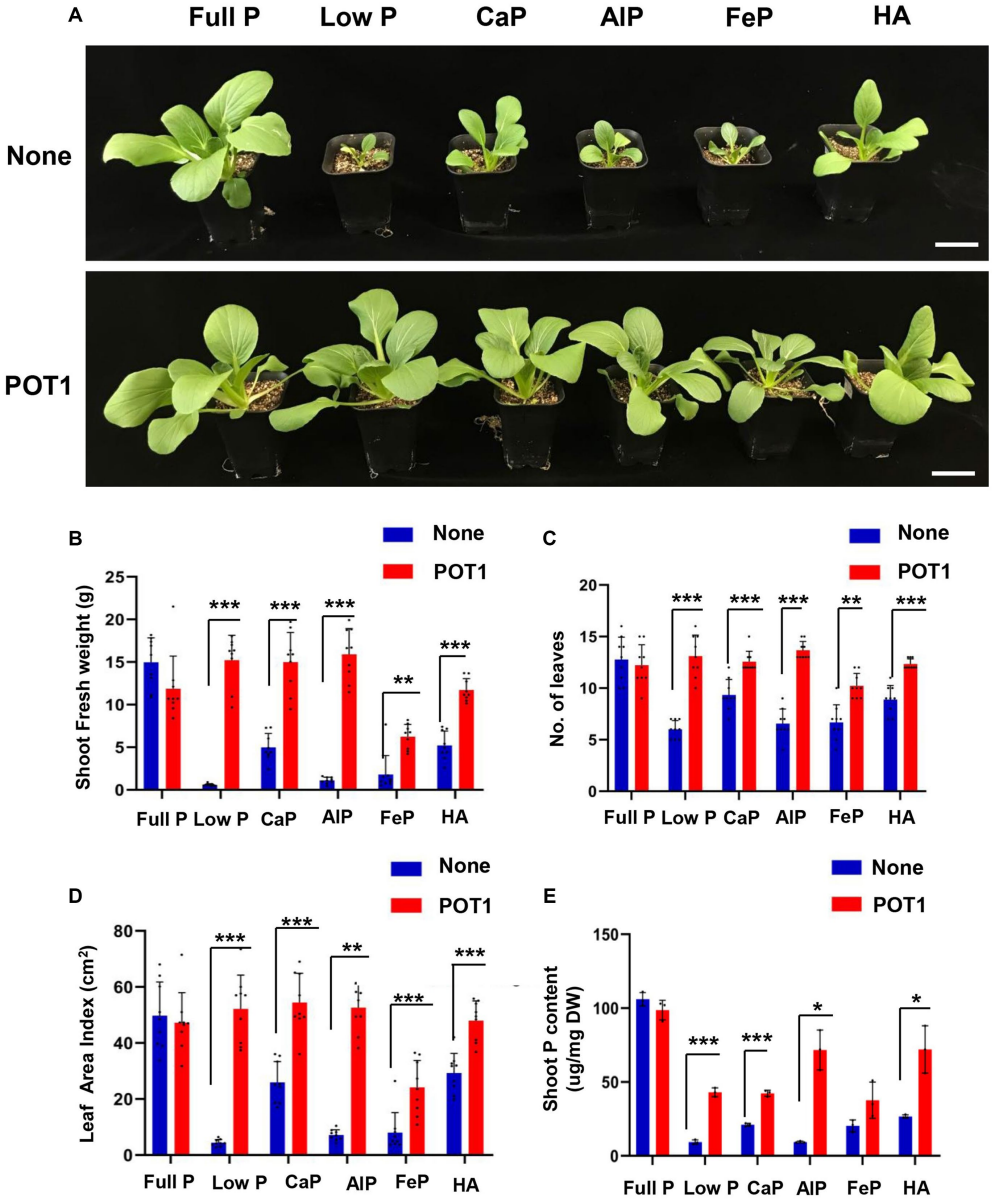
*Penicillium olsonii* TLL1 (POT1) promotes the growth of leafy vegetable Bok Choy under insoluble phosphate and phosphate-limiting conditions. **(A)** A representative image of Bok Choy plants grown in full P, low P, CaP, AlP, FeP, and HA as the sole source of P with and without POT1. Ten-days-old Bok Choy plants were transferred to nutrient-poor vermiculite soil pre-inoculated with and without POT1 and were harvested after 4 weeks. The scale bar represents 5 cm. The bar diagrams show **(B)** shoot fresh weight, **(C)** number of leaves, and **(D)** leaf area index of the 4th leaf of Bok Choy plants co-cultivated with POT1 in full P, low P, CaP, AlP, FeP, and HA conditions after 4 weeks. The combined data from nine independent experiments were analyzed (*n* = 9; unpaired two-tailed *t*-test, ^***^p < 0.001, ^**^p < 0.01, and ^*^p < 0.05). **(E)** Shoot phosphate content of Bok Choy plants co-cultivated with and without POT1 after 4 weeks (*n* = 3; unpaired two-tailed *t*-test, ^**^p < 0.01).

We also examined whether POT1 colonization could promote the growth of leafy vegetable Bok Choy (*B. rapa* subsp. *chinensis*) under different phosphate sources by growing it in vermiculite (Figure 6A and Supplementary Table S12). After 4 weeks, Bok Choy plants grown in low P, tricalcium phosphate (CaP), aluminum phosphate (AlP), iron phosphate (FeP), and hydroxyapatite (HA) conditions without POT1 exhibited stunted growth. In contrast, the biomass of POT1-inoculated plants increased as indicated by fresh shoot weight, number of leaves, and leaf area index (Figures 6B–D; Supplementary Figure S13). Moreover, the total phosphate content was higher in all the inoculated plants under insoluble phosphate and low phosphate conditions, compared with non-inoculated Bok Choy (Figure 6E). When no P condition was simulated in vermiculite soil, the shoot fresh weight, number of leaves, and leaf area index of plants increased in plants treated with POT1 compared to non-inoculated plants (Supplementary Figure S14). However, the biomass of the plants still decreased 7-fold compared to plants grown under full P conditions (Supplementary Table S13).

### 3.9. Growth promotion by POT1 under local soil conditions

We investigated whether POT1 functions in the local soil at Lim Chu Kang in Singapore on Bok Choy and Rice growth. Firstly, we analyzed the pH and macro/micro-nutrient content of the local soil. Our results showed that the pH of Lim Chu Kang soil was 6.5 higher than the pH of the commercial soil (5.7) (Supplementary Figure S15A), which was in the range of highest phosphate availability (Havlin, 2020) (Supplementary Figure S15B). Additionally, the phosphate content of the commercial soil was 4-fold higher than that of Lim Chu Kang soil (Supplementary Figure S15C). However, the phosphate content in Lim Chu Kang soil (50 mg/kg) indicated that they were still sufficient for plant growth (Philip et al., 2021). We also analyzed other macro/micro-nutrients such as nitrogen, potassium, calcium, magnesium, iron, sodium, copper, manganese, zinc, and boron in the commercial and Lim Chu Kang soils (Supplementary Figures S16A,B). Notably, the content of copper, manganese, and zinc in Lim Chu Kang soil was higher than that of the commercial soil (Supplementary Table S14).

**Figure 7:**
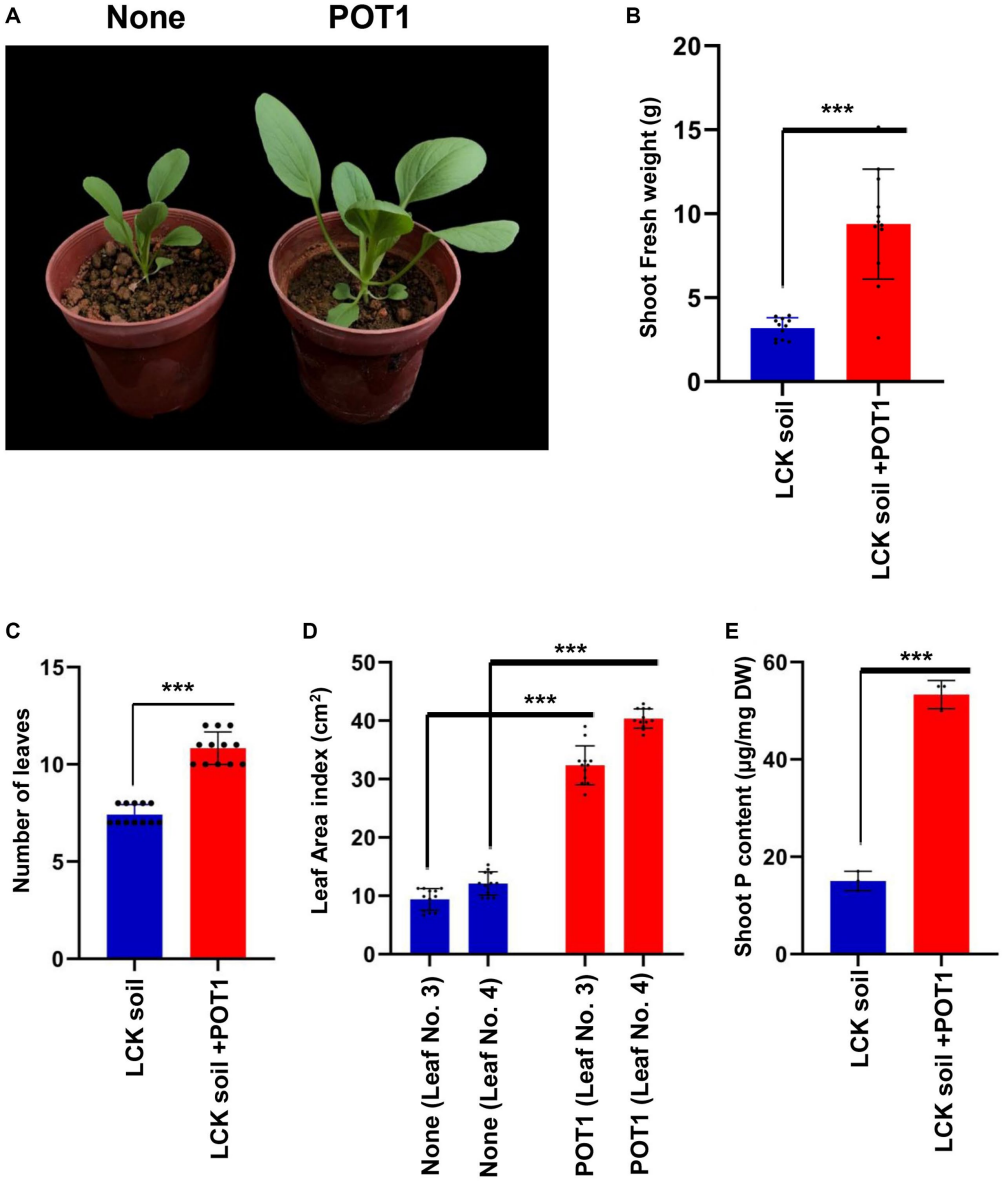
*Penicillium olsonii* TLL1 (POT1) promotes the plant growth index of leafy vegetables in Singapore’s local soil. **(A)** A representative image of Bok Choy plants grown in Lim Chu Kang soil with and without POT1. Ten-days-old Bok Choy plants were transferred to Lim Chu Kang soil pre-inoculated with and without POT1 and harvested after 4 weeks. The scale bar represents 5 cm. The bar diagrams show **(B)** shoot fresh weight, **(C)** the number of leaves, and **(D)** the leaf area index of the 3rd and 4th leaves of Bok Choy plants co-cultivated with POT1 after 4 weeks. The combined data from 12 independent experiments are represented (*n* = 12; unpaired two-tailed *t*-test, ^***^p < 0.001, ^**^p < 0.01, and ^*^p < 0.05). **(E)** Shoot phosphate content of Bok choy plants co-cultivated with and without POT1 after 4 weeks (*n* = 3; unpaired two-tailed *t*-test, ^**^p < 0.01).

**Figure 8:**
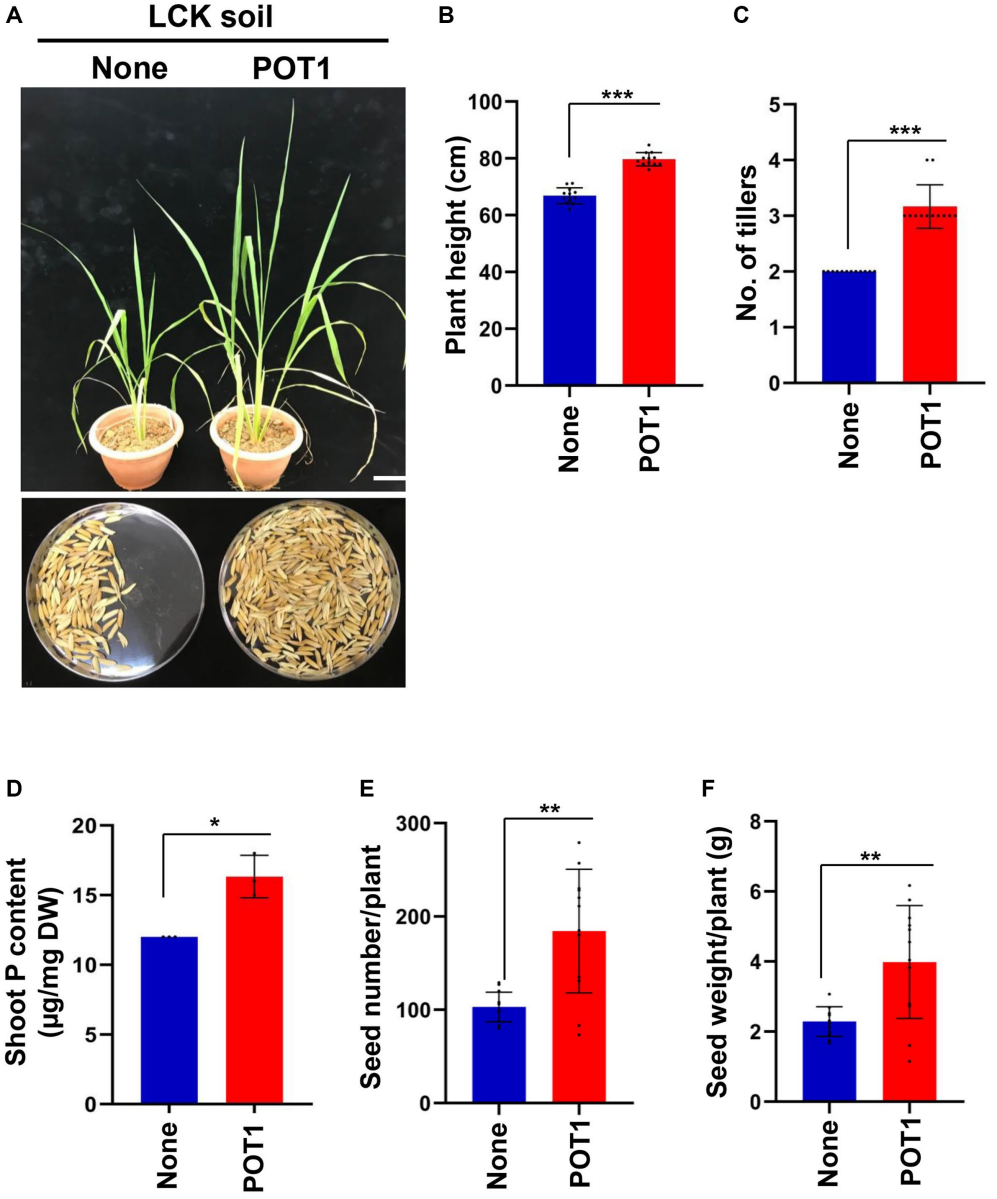
*Penicillium olsonii* TLL1 (POT1) promotes the plant growth index of rice in Singapore’s local soil. **(A)** A representative image of rice grown in Lim Chu Kang soil with and without POT1 after 6 weeks. The scale bar represents 10 cm. Fourteen-days-old Rice seedlings were transferred to LCK soil pre-inoculated with and without POT1 and were grown for 3 months. The bar diagrams show **(B)** plant height, **(C)** number of tillers, **(D)** shoot phosphorus content after 6 weeks, **(E)** seed number per plant, and **(F)** seed weight per plant co-cultivated with and without POT1 in LCK soil. The combined data from 12 independent experiments were analyzed (*n* = 12; unpaired two-tailed *t*-test, ^***^p < 0.001, ^**^p < 0.01, and ^*^p < 0.05).

For 4 weeks, we grew Bok Choy plants in Lim Chu Kang soil with/without POT1 (Supplementary Figure S17). We found that POT1 improved the growth conditions of the plants, as indicated by the increase in shoot fresh weight, number of leaves, and leaf index in POT1 inoculated plants (Figures 7A–D and Supplementary Table S15). Moreover, the total phosphorus content in the leaves of POT1-inoculated plants was higher than that of non-inoculated plants (Figure 7E and Supplementary Table S15). We also confirmed that POT1 promoted the growth of Rice, a monocot plant, in Lim Chu Kang soil (Figure 8; Supplementary Figure S18). The co-cultivation of Rice plants with POT1 resulted in increased plant height (Figures 8A,B), number of tillers (Figure 8C), and shoot phosphate content (Figure 8D) compared with plants grown without POT1 inoculation. Furthermore, the increased tiller number was accompanied by an increase in seed number (Figure 8E), and seed dry weight (Figure 8F) was also increased by POT1 inoculation (Supplementary Table S16).

In addition, we tested the effect of POT1 on plant growth under Lim Chu Kang soil conditions using heat-killed POT1 as a negative control. We found that shoot fresh weight, number of leaves, and leaf area index of Bok Choy were significantly higher in live POT1 inoculated plants compared to heat-killed POT1 and uninoculated plants (Supplementary Figure S19 and Supplementary Table S17). Similarly, in the Rice growth test, heat-killed POT1 was non-functional, as indicated by shorter plant height, lower tiller numbers, and reduced seed weight/number compared with live POT1 (Supplementary Figure S20 and Supplementary Table S18). These results suggest that the phosphate-solubilizing capability of POT1 helps plants absorb phosphate sources from soils containing insoluble phosphate, thereby increasing plant growth.

### 3.10. Comparison of five *Penicillium* strains on phosphate solubilizing activity and Arabidopsis growth

The capability of POT1 to solubilize tricalcium phosphate was evaluated against other *Penicillium* strains including *P. bilaiae*, *P. chrysogenum*, *P. janthinellum*, and *P. simplicissimum* (Supplementary Figure S21). In mediums containing CaP, the soluble phosphate concentrations for *P. chrysogenum*, *P. janthinellum*, and *P. simplicissimum* were measured at 1.69 mg/mL, 1.84 mg/mL, and 1.97 mg/mL, respectively. The highest solubilizing activity was shown in *P. bilaiae* (3.44 mg/mL), followed by POT1 (3.06 mg/mL) (Supplementary Table S19). Based on these results, growth promotion activity was also compared between POT1 and the aforementioned *Penicillium* strains under phosphate-deficient conditions (Supplementary Figure S22A). All strains showed longer primary root lengths compared to the control, which was without any fungal inoculation. Among the five *Penicillium* strains, plants inoculated with POT1 had the longest primary root length (Supplementary Figure S22B). Similarly, shoot fresh weight in plants treated with POT1 (a 2-fold increase) was the highest among all the strains (Supplementary Figure S22C). Unexpectedly, no difference in shoot fresh weight was found between plants treated with *P. simplicissimum* and control plants. Moreover, in the case of plants co-cultivated with *P. janthinellum*, a 2.56-fold decrease in shoot fresh weight was observed. Regarding root fresh weight, all inoculated plants exceeded the non-inoculated controls, with *P. chrysogenum* demonstrating the highest increase (Supplementary Figure S22D and Supplementary Table S20).

To investigate plant growth by the phosphate solubilizing activity of each fungus, we tested Arabidopsis growth in vermiculite using an insoluble phosphate source, which is calcium phosphate (CaP) (Supplementary Figure S23A). The shoot fresh weight of Arabidopsis inoculated with POT1 was higher than that of other strains under these conditions (Supplementary Figure S23B). Furthermore, we measured anthocyanin accumulation, which is a typical phosphate deficiency response in plants. The lowest anthocyanin content was observed in plants inoculated with POT1 and *P. simplicissimum* (Supplementary Figure S23C and Supplementary Table S21). Overall, these results indicate that POT1 demonstrated superior activity in comparison to the other *Penicillium* strains.

## 4. Discussion

In our study, we identified that the newly isolated fungal strain, *P. olsonii* TLL1 (POT1), has a high ability to solubilize phosphate and promote root elongation in Arabidopsis (Figure 1). Even if the phosphate source is completely omitted from the growth medium, the length of the primary root is longer with POT1 inoculation. Previously, several *Penicillium* spp. were isolated from the greenhouses of a botanical garden and *P. olsonii* was identified to be one of the predominant airborne fungal species (Rodolfi et al., 2003). Recently, the biocontrol activity and plant growth-promoting potential of *P. olsonii* strains isolated from different environments have also been revealed (Rojas et al., 2022; Tarroum et al., 2022). Here, we demonstrate the growth-promoting potential of POT1 especially under phosphate limiting conditions.

The STOP1 transcription factor is one of the crucial checkpoints in root development to phosphate deficiency stress in Arabidopsis. Our study shows that the STOP1 protein level is decreased by POT1 inoculation under full P and low P conditions, implying that the reduction of STOP1 protein leads to a low ALMT1 transcript level (Figures 2, 3A). STOP1 stability is reported to be post-translationally regulated by RAE1, an E3 ubiquitin ligase, thereby controlling primary root length and *STOP1* mutants, and RAE1 overexpressed plants have a long primary root under phosphate deficiency (Sawaki et al., 2009; Balzergue et al., 2017; Zhang et al., 2019). A recent report demonstrated that in Arabidopsis, the premature leaf senescence induced by the soil-borne fungus *Verticillium dahliae* is influenced by the protein elicitor PevD1, which is secreted by *V. dahliae*. When PevD1 is overexpressed or transferred by *V. dahliae*, it not only accelerates leaf senescence but also targets and stabilizes the senescence-related NAC transcription factor ORE1. This happens by interfering with ORE1’s interaction with the RING-type ubiquitin E3 ligase NLA, leading to increased ethylene production and enhanced senescence (Zhang et al., 2021). Similarly, more research is needed to identify specific elicitors that affect the degradation of STOP1 by RAE1.

Moreover, we confirmed that POT1 inhibited callose and NO accumulation in root cells under phosphate deficiency (Figures 3B,C). As negative factors of primary root elongation inhibiting cell-to-cell communication, callose deposition and NO are accumulated in the root apical meristem and entire root cells, respectively.

Despite being grown under low phosphate conditions, Arabidopsis’ roots elongate normally due to the inhibition of callose and NO accumulation with root colonization of POT1. These metabolites can be degraded and scavenged by beta-1,3 glucanases and flavonoids, respectively (Wang et al., 2010; Burch-Smith and Zambryski, 2012; Duarte et al., 2014; Muller et al., 2015).

The secretion of β-1,3 glucanases by endophytic fungi is well documented as an antagonistic activity against invading fungal pathogens (Markovich and Kononova, 2003). Endophytic fungal communities with phosphate solubilizing activity associated with the rhizosphere of healthy maize and rice plants secrete β-1,3 glucanases, chitinases, and amylases (Potshangbam et al., 2017). Besides, *Aspergillus nidulans* and *Aspergillus oryzae* are reported to secrete flavonoids in cultures (Qiu et al., 2010). Taken together, we suggest that POT1 might control STOP1 protein stability and secrete beta-1,3 glucanases and flavonoids in low-phosphate environments. Thus, further studies are required to confirm the mechanism of post-translational regulation as well as the secretion of beta-1,3 glucanases and flavonoids by POT1 under phosphate-limiting conditions. Therefore, further study is required to determine whether POT1 can secrete and transfer a particular flavonoid and beta-1,3-glucanases to plant root cells using secondary metabolite and proteome analysis as well as investigate flavonoid content and beta-1,3-glucanase expression level induced by POT1 inoculation.

Organic acid secretion is the widely reported mechanism of phosphate solubilization, where PSFs break down insoluble phosphates by secreting organic acids onto soil surfaces (Rawat et al., 2021). Malic acid was most competent to solubilize CaP, followed by citric, formic, and succinic acids (Gaind, 2016). It has been reported that tartaric acid and citric acid were dominant in *Penicillium oxalicum* P4, while malic and citric acid dominated in *A. niger* P85 cultures when solubilizing tricalcium phosphate (Yin et al., 2015). The commercial strain, *P. bilaiae*, has been shown to produce oxalic and citric acids (Sanchez-Esteva et al., 2016). We have shown that in low P conditions, POT1 highly exudates gluconic acid while malic acid and citric acid are reduced.

The use of microbial inoculants has gained acceptance over chemical fertilizers as they are eco-friendly and help to improve soil health. The application of fungal species as biofertilizers to arable land to improve soil quality is emerging as an eco-friendly approach in modern agriculture (Kour et al., 2020). *P. bilaiae* was widely used as a biofertilizer, and several studies conducted in growth chambers and the field increased the biomass, phosphate uptake, and grain yield in wheat (*Triticum aestivum* L.) (Sanchez-Esteva et al., 2016), pea (Vessey and Heisinger, 2001), and lentil (Wakelin et al., 2007). Among the *Penicillium* genus, *P. bilaji*, *P. italicum*, *P. albidum*, *P. frequentans*, *P. simplicissimum*, *P. rubrum*, *P. expansum*, *P. oxalicum*, and *P. citrinum* have been commercially used as biofertilizers for the mobilization of P, Mn, Zn, Fe, Co, Cu, and Mo and biotic and abiotic stress tolerance (Odoh et al., 2020). *P. bilaiae* was reported to be effective in calcareous soil compared to moderately acidic soil (Sanchez-Esteva et al., 2016). In our study, we have confirmed that plant growth conditions in Arabidopsis, Bok Choy, and Rice in vermiculite and Singapore’s local soil, which has low phosphate availability, are improved due to the P-solubilizing activity of POT1 (Figures 5–8).

Phosphate fixation in soil depends on edaphic factors such as pH, organic matter content, the presence of exchangeable cations such as Fe, Al, Ca, and Mg, and clay content, which contribute to phosphate insolubility. The phosphate added as agro fertilizer to the soil reacts with cation ions such as Al, Ca, and Fe and becomes fixed in the soil, which is inaccessible for uptake by the plant roots (Penn and Camberato, 2019). Hence, phosphate-solubilizing fungi (PSF) could be widely employed as a potential solution to improve phosphate uptake and use efficiency.

Phosphate-solubilizing fungi thrive in the rhizosphere and establish symbiotic associations with the root systems of plants. Endophytic fungi such as *Penicillium*, *Aspergillus*, *Trichoderma*, *Piriformospora*, and *Curvularia* are among the active PSF species involved in phosphate cycling (Mehta et al., 2019). The *P. guanacastense* JP-NJ2 strain has a high ability to solubilize aluminum phosphate (Qiao et al., 2019). The solubilization of recalcitrant phosphate sources by *Aspergillus* spp. and *Penicillium* spp. generally decreased the liquid medium pH for the solubilization of insoluble phosphate (Acevedo et al., 2014). Here, we confirmed the phosphate-solubilizing activity of POT1 in insoluble phosphate complexes such as tricalcium phosphate, aluminum phosphate, iron phosphate, and hydroxyapatite (Figure 4). Among these, the highest solubilizing capability is observed in culture media containing tricalcium phosphate (Figure 4D). In addition, we showed that POT1 was remarkably effective on root growth promotion and phosphate solubilizing activity in the phosphate-deficient medium and insoluble phosphate-contained soil when compared to other *Penicillium* strains such as *P. bilaiae*, *P. chrysogenum*, *P. janthinellum*, and *P. simplicissimum*. Thus, we suggest that POT1, which showed better activity than other *Penicillium* strains, can be used as a high-potential biofertilizer for both monocot and dicot crop growth as well as the different types of soil conditions, which are upland and paddy soil. Furthermore, combinations of various beneficial fungal strains are required for the evaluation of plant growth and viability tests among fungal strains. Overall, this study provides useful information for developing more efficient biofertilizers than the existing ones.

## 5. Conclusion

The optimal usage of chemical fertilizers is necessary to maintain ecosystem function and develop sustainable agriculture. Thus, application, research, and methodology developments of biofertilizers need to be emphasized on improving effective, stable, and non-toxic microbiome strains for promoting plant growth. Using POT1, a potential biofertilizer, our study provides information on the combined action of both phosphate-solubilizing function and inhibitor removal of primary root elongation, especially under phosphate-limiting conditions. Therefore, these findings will support the development of biofertilizers for improved phosphate use efficiency and apply to different types of plants, such as monocot and dicot plants, increasing the yield even under low or no phosphate conditions as well as different conditions of soil, such as upland and paddy soils. Further study is required to investigate the functions and changes of diversification in the soil ecosystem due to biotic interactions between soil microbiomes and POT1. Specifically, research should explore combinations of POT1 with other beneficial fungi and bacteria to identify microbiome combinations that maximize growth promotion and yield in crops. Moreover, in our study, there is limited data on the effects of POT1 on plant growth in acidic conditions and different soil textures. Further research is needed to explore the functions of POT1 under various field conditions. Taken together, our research would contribute toward a better solution for sustainable agriculture with reduced fertilizer cost, especially phosphate, increasing its use efficiency and increasing crop yield.

## Data availability statement

The original contributions presented in the study are included in the article/Supplementary material, further inquiries can be directed to the corresponding author.

## Author contributions

ES: Formal analysis, Investigation, Methodology, Project administration, Writing – original draft, Writing – review & editing. VA: Investigation, Writing – original draft, Writing – review & editing. SD: Formal analysis, Methodology, Writing – review & editing. YS: Formal analysis, Investigation, Visualization, Writing – review & editing. SL: Investigation, Writing – review & editing. NN: Supervision, Writing – review & editing. RS: Supervision, Writing – review & editing. ZY: Supervision, Writing – review & editing. BP: Conceptualization, Data curation, Formal analysis, Funding acquisition, Methodology, Project administration, Resources, Supervision, Validation, Visualization, Writing – original draft, Writing – review & editing.

## Footnotes

^1^ https://imagej.nih.gov/ij/download/
