# Supplementary material for: Plant growth promotion under phosphate deficiency and improved phosphate acquisition by new fungal strain, Penicillium olsonii TLL1
Source: Front Microbiol. 2023 Oct 19;14:1285574. doi: 10.3389/fmicb.2023.1285574 (PMC10642178; doi:10.3389/fmicb.2023.1285574)
Supplement: Supplementary file 1 [file Data_Sheet_1.docx]

**Supplementary Figures**


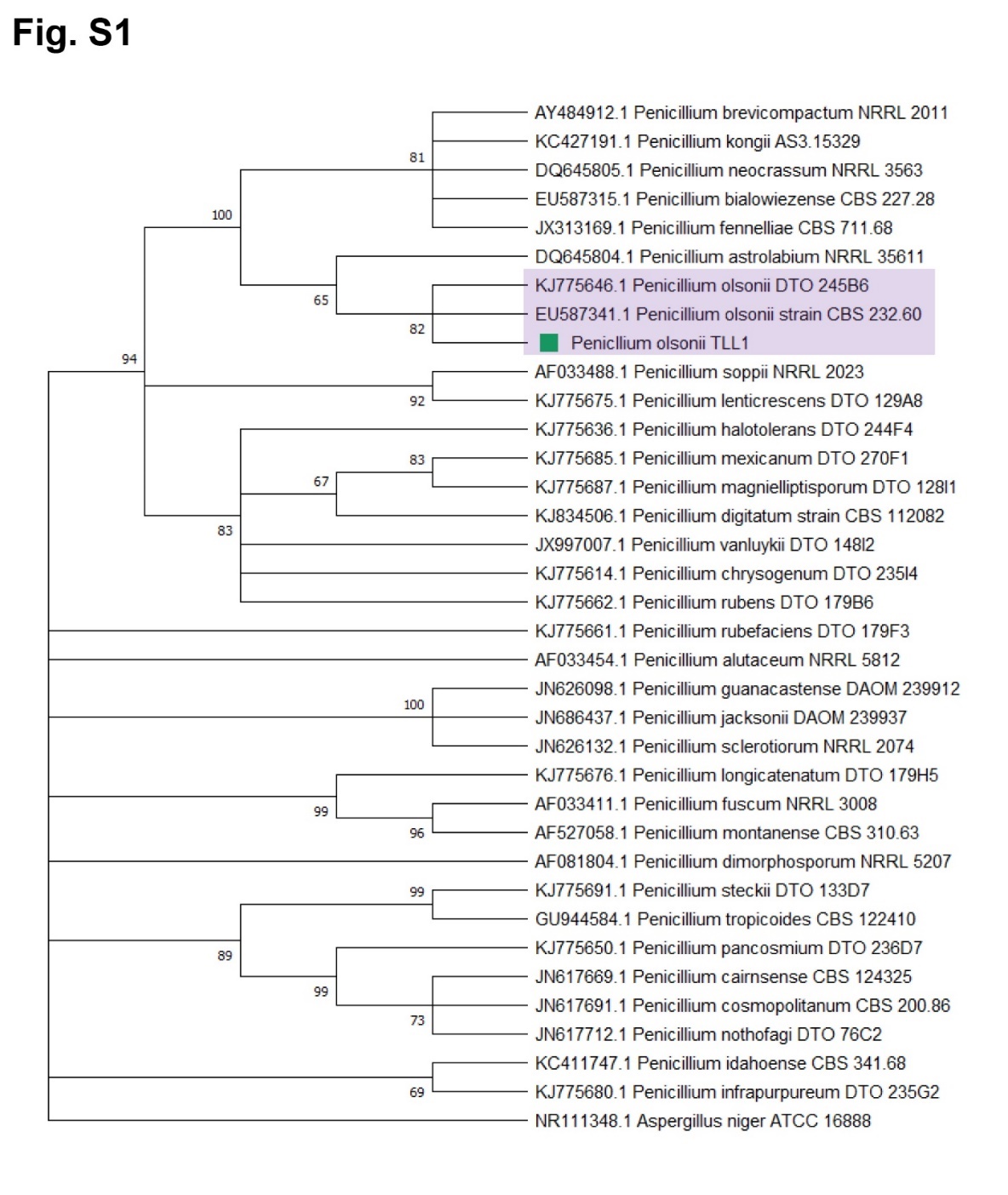


**Supplementary Figure S1.** Maximum-likelihood tree of the phosphate solubilizing fungus, *P. olsonii* TLL1 (POT1) based on *ITS* sequences. Best-scoring Maximum Likelihood (ML) tree using Tamura Nei model of MEGA7 based on partial internal transcribed spacer (*ITS*) sequences, showing the clustering of POT1 with other *Penicillium* strains. The bootstrap values of the ML analysis are presented at the nodes.


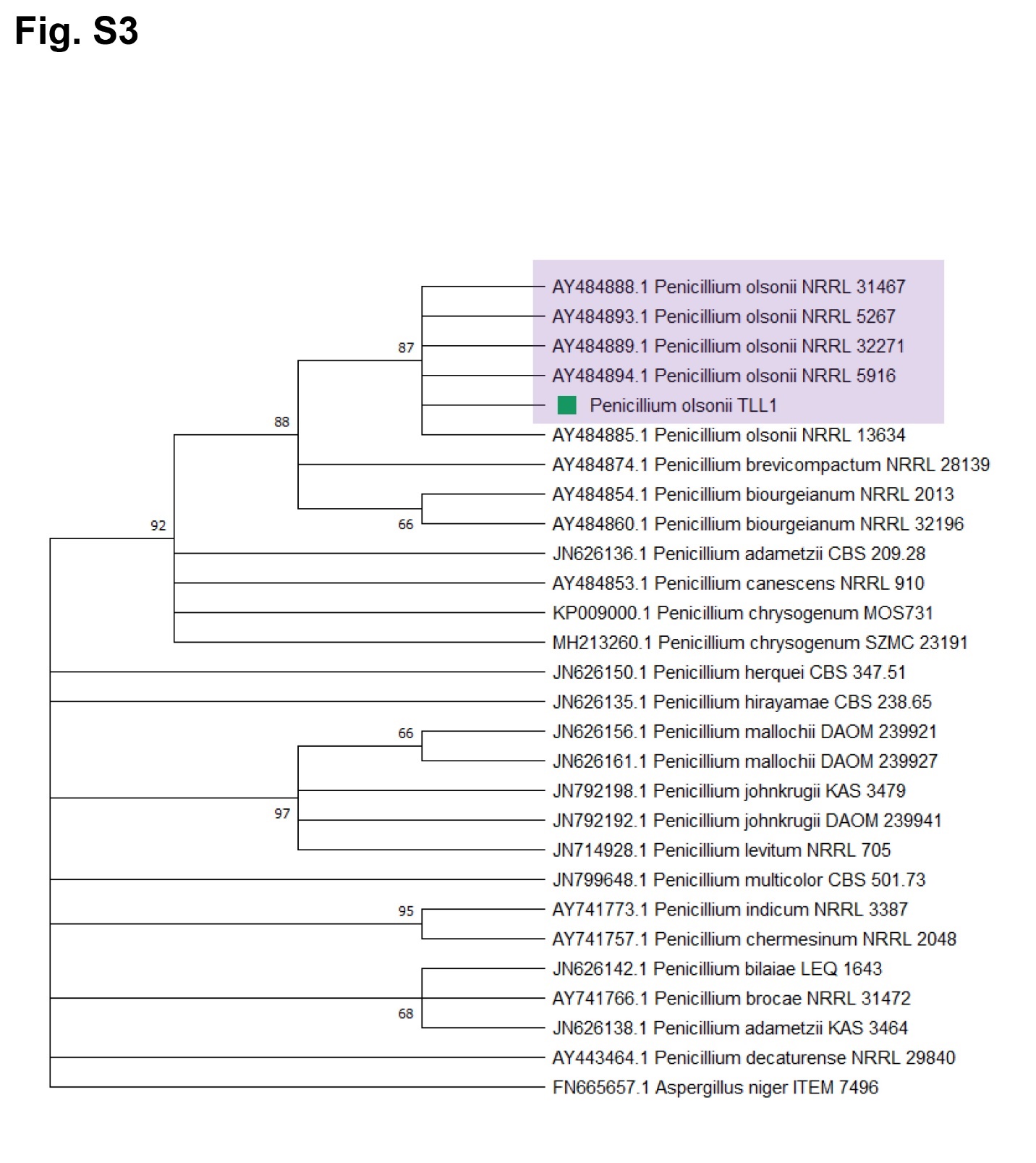


**Supplementary Figure S2.** Maximum-likelihood tree of the phosphate solubilizing fungus, *P. olsonii* TLL1 (POT1) based on translation elongation factor-1-α (*TEF1-α*) sequences. Best-scoring Maximum Likelihood (ML) tree using Tamura Nei model of MEGA7 based on partial translation elongation factor-1-α (*TEF1-α*) sequences, showing the clustering of POT1 with other *Penicillium* strains. The bootstrap values of the ML analysis are presented at the nodes.


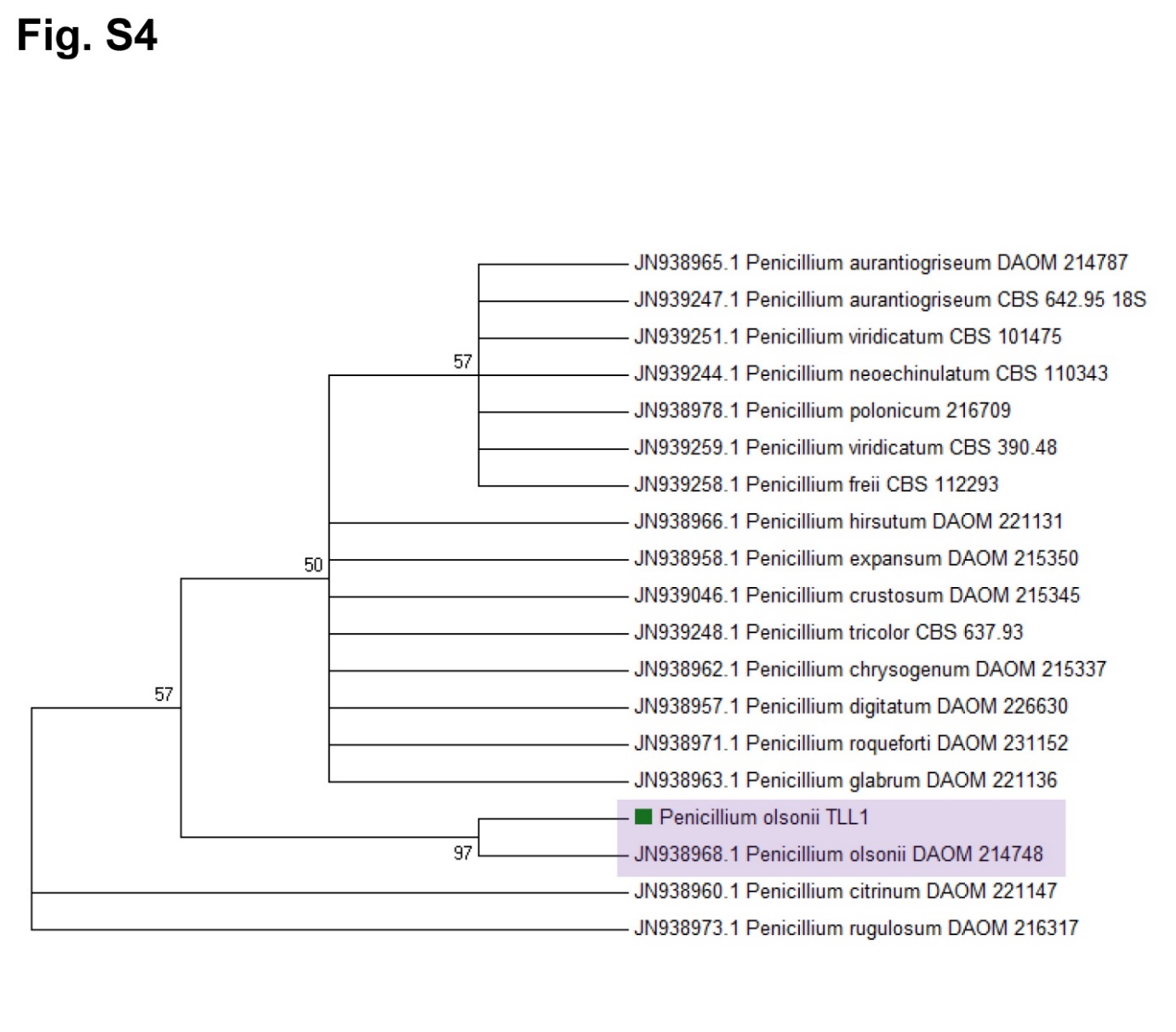


**Supplementary Figure S3.** Maximum-likelihood tree of the phosphate solubilizing fungus, *P. olsonii* TLL1 (POT1) based on Small ribosomal subunit (*SSU*) sequences. Best-scoring Maximum Likelihood (ML) tree using Tamura Nei model of MEGA7 based on partial Small ribosomal subunit (*SSU*) sequences, showing the clustering of POT1 with other *Penicillium* strains. The bootstrap values of the ML analysis are presented at the nodes.


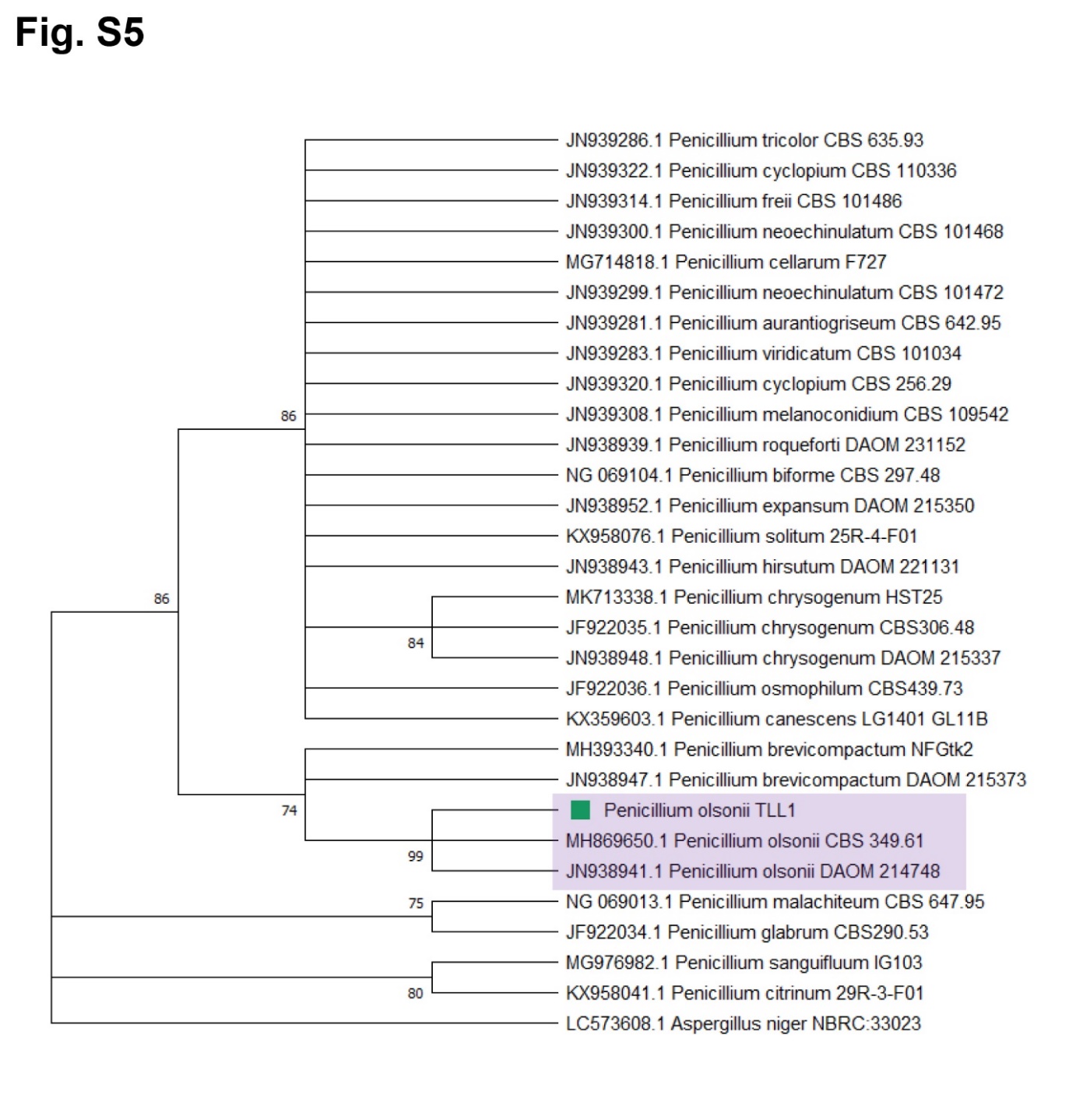


**Supplementary Figure S4.** Maximum-likelihood tree of the phosphate solubilizing fungus, *P. olsonii* TLL1 (POT1) based on Large ribosomal subunit (*LSU*) sequences. Best-scoring Maximum Likelihood (ML) tree using Tamura Nei model of MEGA7 based on partial large ribosomal subunit (*LSU*) sequences, showing the clustering of POT1 with other *Penicillium* strains. The bootstrap values of the ML analysis are presented at the nodes.


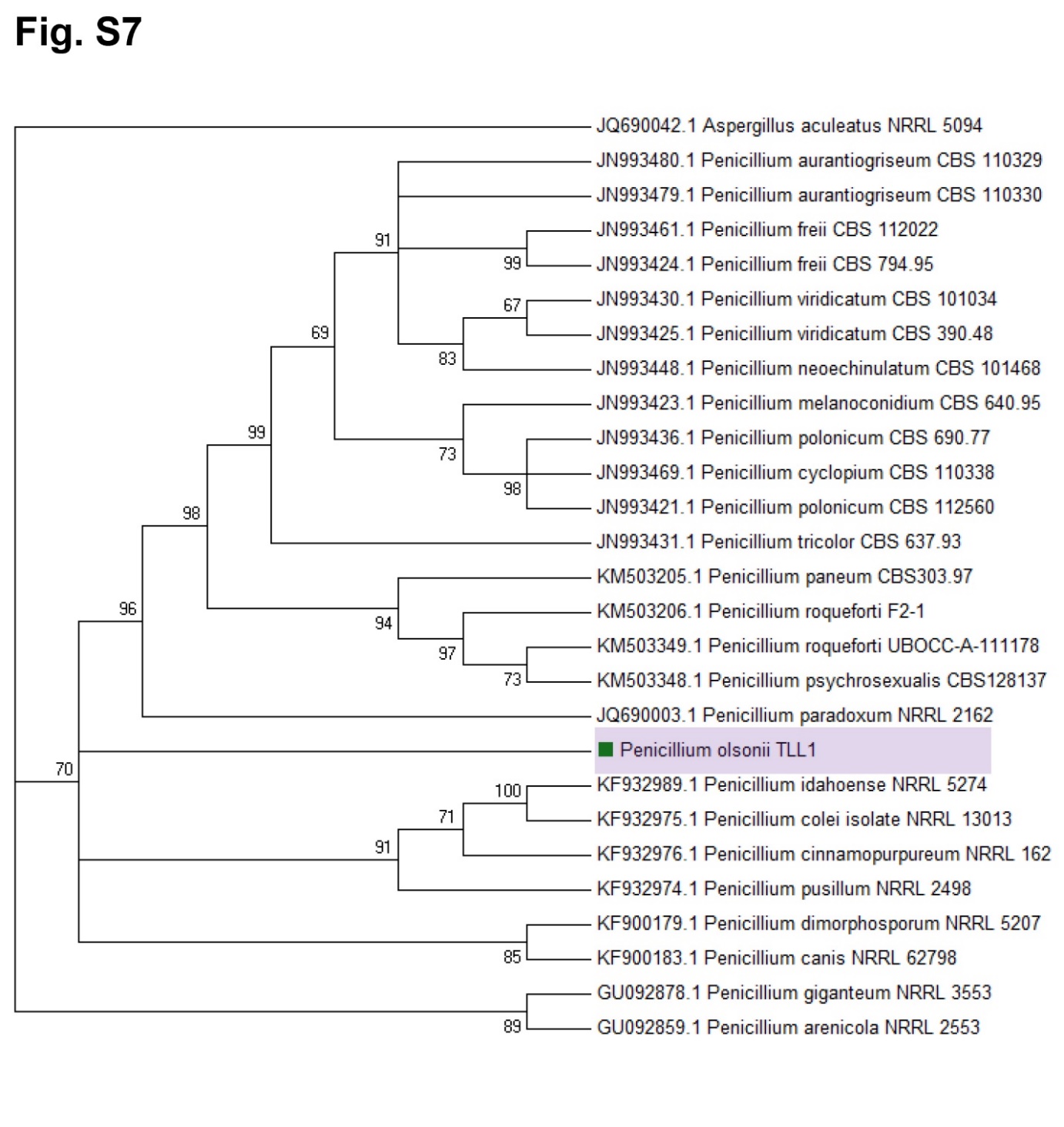


**Supplementary Figure S5.** Maximum-likelihood tree of the phosphate solubilizing fungus, *P. olsonii* TLL1 (POT1) based on Mini-chromosome maintenance protein (*MCM7*) sequences. Best-scoring Maximum Likelihood (ML) tree using Tamura Nei model of MEGA7 based on partial Mini-chromosome maintenance protein (*MCM7*) sequences, showing the clustering of POT1 with other *Penicillium* strains. The bootstrap values of the ML analysis are presented at the nodes.


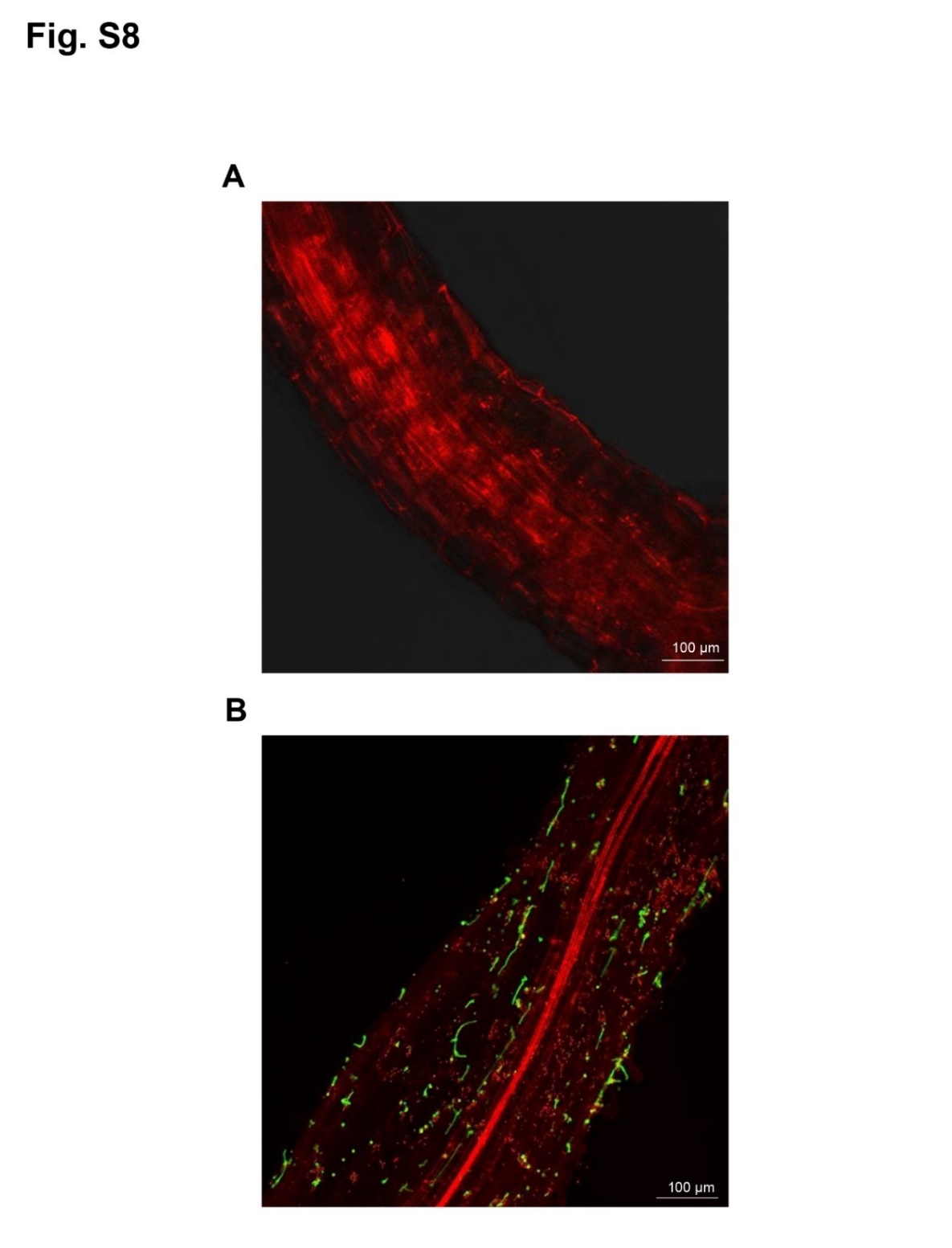


**Supplementary Figure S6.** Colonization of roots by *P. olsonii* TLL1 (POT1) showing hyphal growth along the root tissue. Representative images of WGA-AF488 and PI co-stained Arabidopsis roots **(A)** uninoculated and **(B)** inoculated with POT1. Arabidopsis root samples were harvested after 10 days of co-cultivation with POT1 in full P media. Fungal hyphae were stained with WGA-AF488 (green), and plant cell walls (red) were stained with propidium iodide.


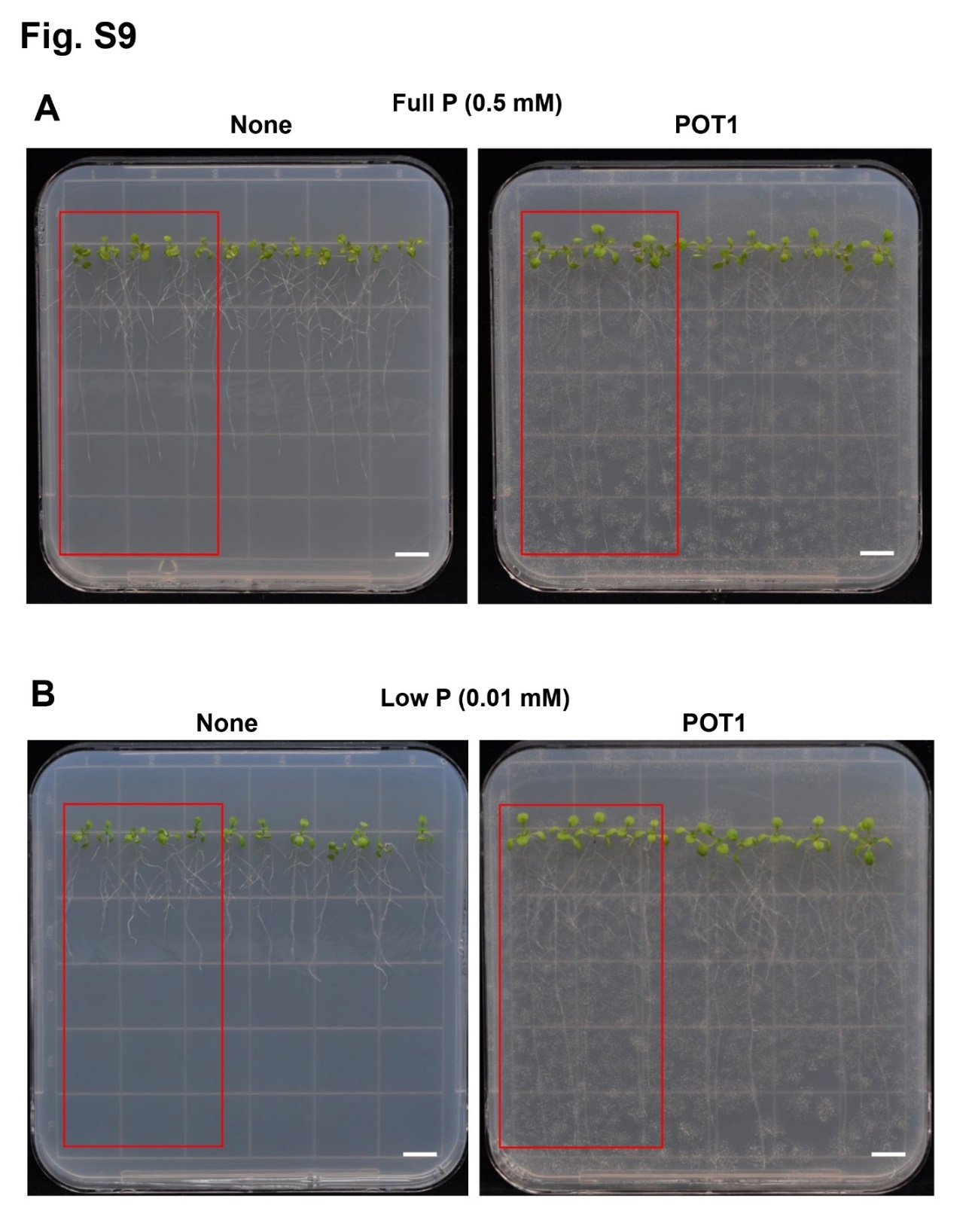


**Supplementary Figure S7.** *P. olsonii* TLL1 (POT1) mediated plant growth promotion of Arabidopsis under **(A)** P sufficient and **(B)** P-limiting conditions, Related to Figures 1C and 1H. Four-day-old Arabidopsis WT, Col-0 seedlings were transferred to full P and low P media precultured with and without POT1. Root lengths were measured after ten days of co-cultivation. Scale bar represents 1 cm.


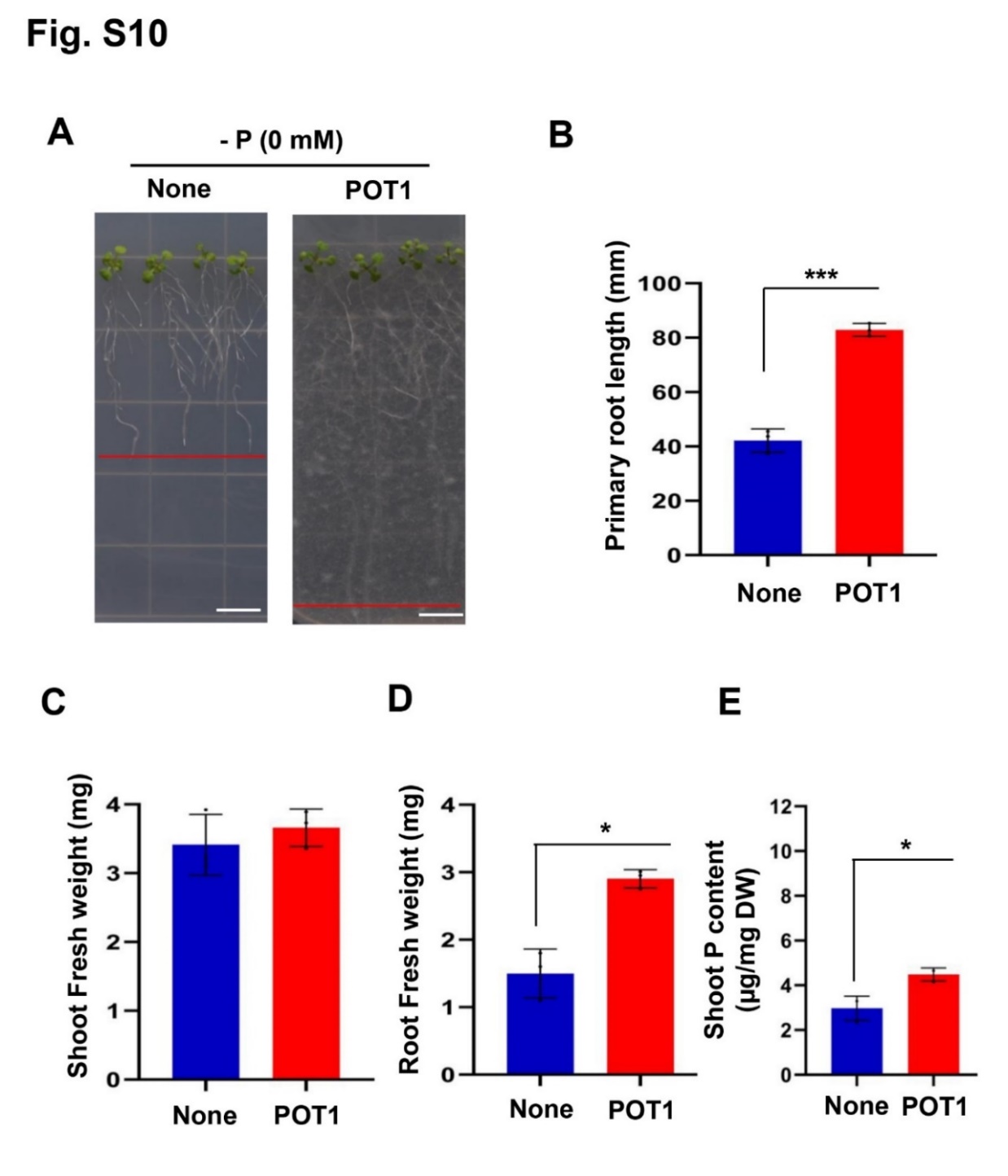


**Supplementary Figure S8.** *P. olsonii* TLL1 (POT1) elongates primary roots of Arabidopsis without any soluble P source. A representative image of Arabidopsis plants grown in -P conditions with and without POT1. **(A)** Four-day-old Arabidopsis plants were transferred to -P media inoculated with and without POT1 under *in vitro* conditions for 10 days. The bar diagrams show **(B)** primary root length, **(C)** Shoot fresh weight, **(D)** Root fresh weight, and **(E)** shoot P content of Arabidopsis WT plants co-cultivated with POT1 in no P conditions. The combined data from ten independent experiments is shown (n=10; unpaired two-tailed t-test, ****P* <0.001; ***P* <0.01; **P* <0.05). Scale bar represents 1 cm.


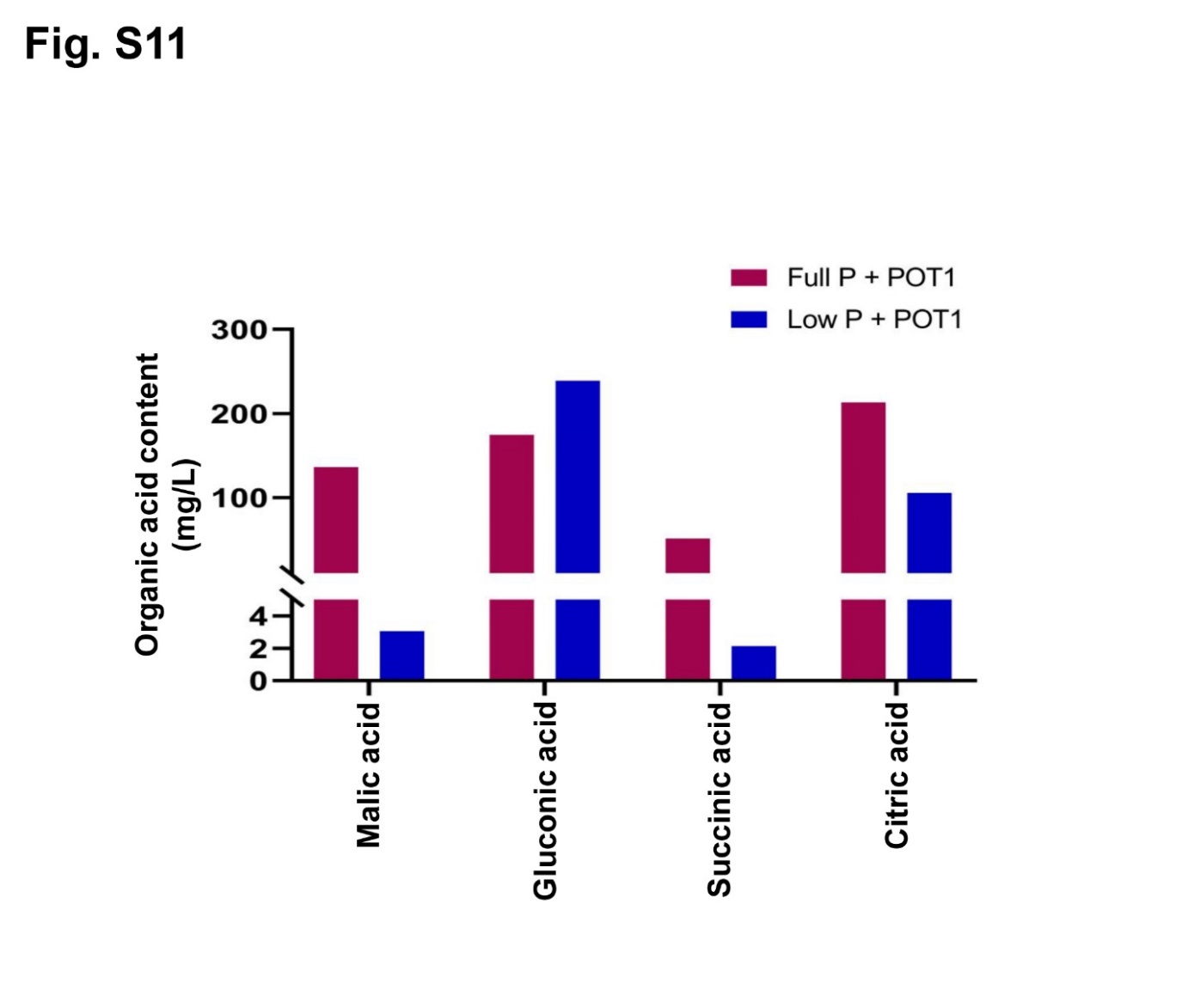


**Supplementary Figure S9.** Organic acids secreted by *P. olsonii* TLL1 (POT1) under P-sufficient and P-limiting conditions. A bar diagram representing the concentration of organic acids such as malic acid, gluconic acid, succinic acid and citric acid secreted by POT1 when cultured under full P and low P conditions for seven days.


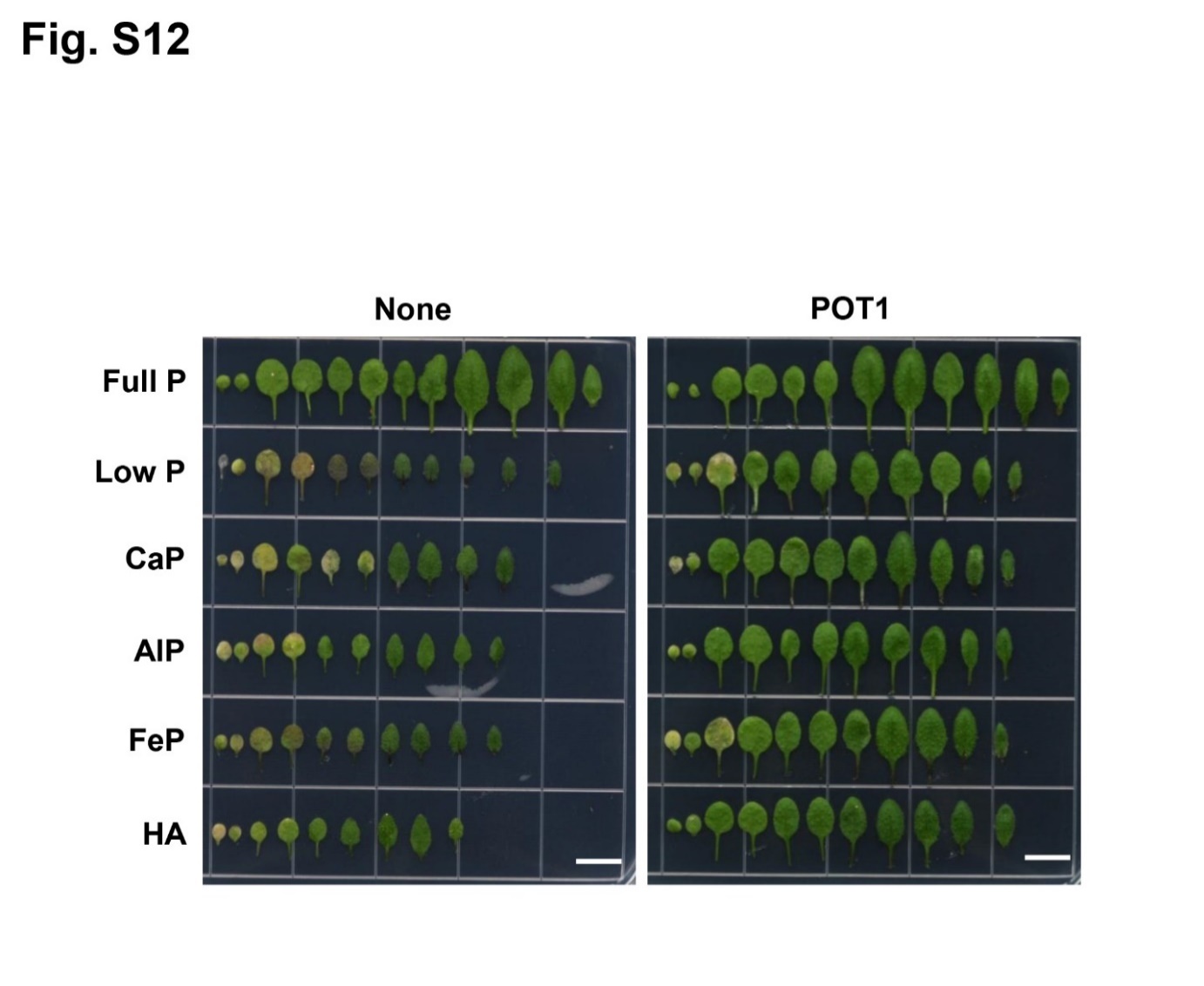


**Supplementary Figure S10.** *P. olsonii* TLL1 (POT1) improves the number of leaves of Arabidopsis WT Col-0 when cultured under insoluble phosphate sources, P-sufficient and P-limiting conditions, Related to Figure 5. A representative image of *A. thaliana* WT leaves when grown in full P, low P, CaP, AlP, FeP and HA as sole source of P with and without POT1.


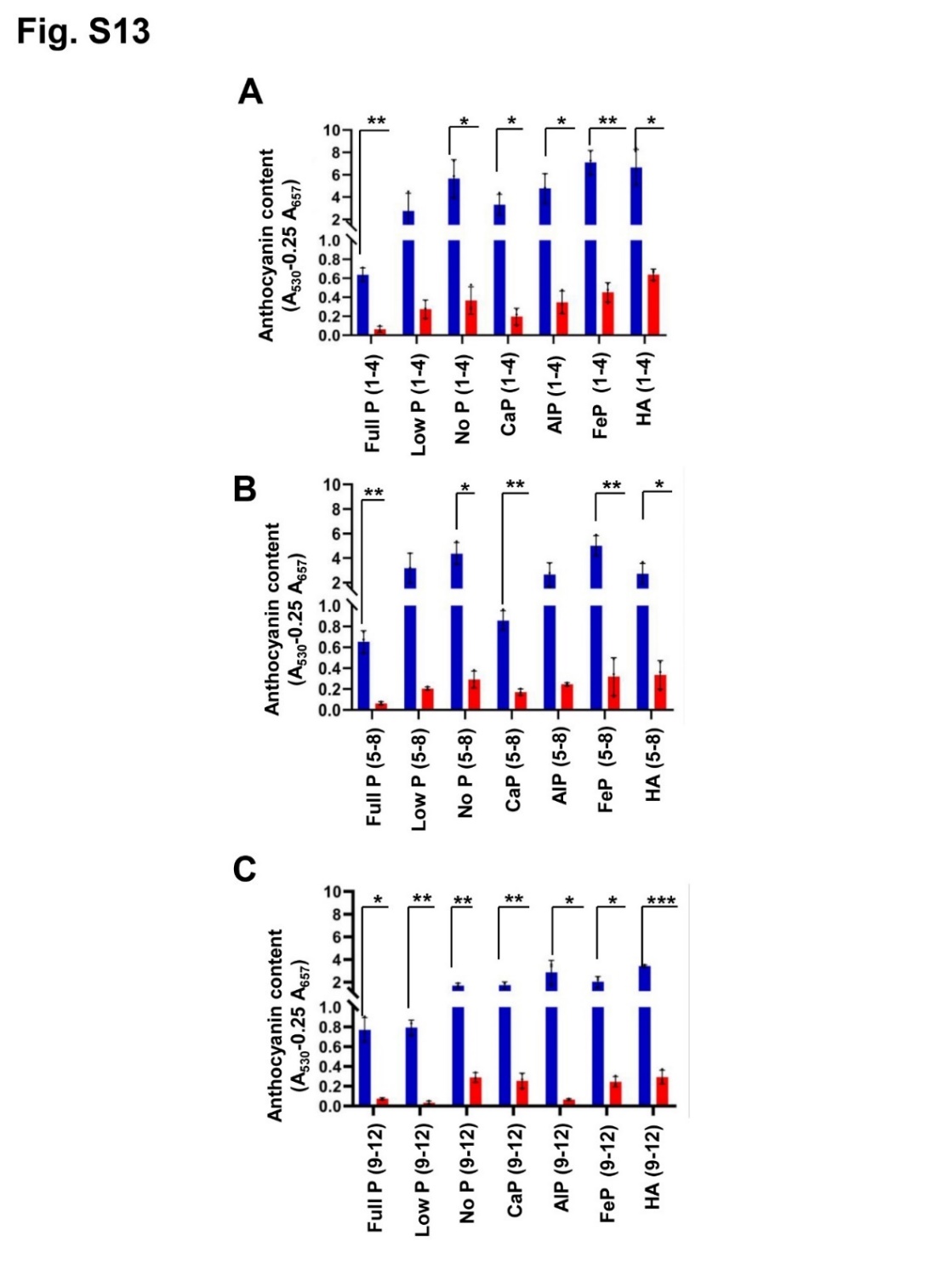


**Supplementary Figure S11.** *P. olsonii* TLL1 (POT1) reduces anthocyanin accumulation in the leaves of Arabidopsis under P-sufficient and P-limiting conditions. Bar diagrams representing anthocyanin content in Arabidopsis plants when grown under different phosphate sources with and without POT1 inoculation in vermiculite soil. Relative anthocyanin content per gram fresh weight were represented in **(A)** 1-4 leaves, **(B)** 5-8 leaves, and **(C)** 9-12 leaves of Arabidopsis grown under full P, low P, No P, CaP, AlP, FeP and HA as sole source of P with and without POT1 in three independent experiments (n=3; unpaired two-tailed t-test, ****P* <0.001; ***P* <0.01; **P* <0.05).


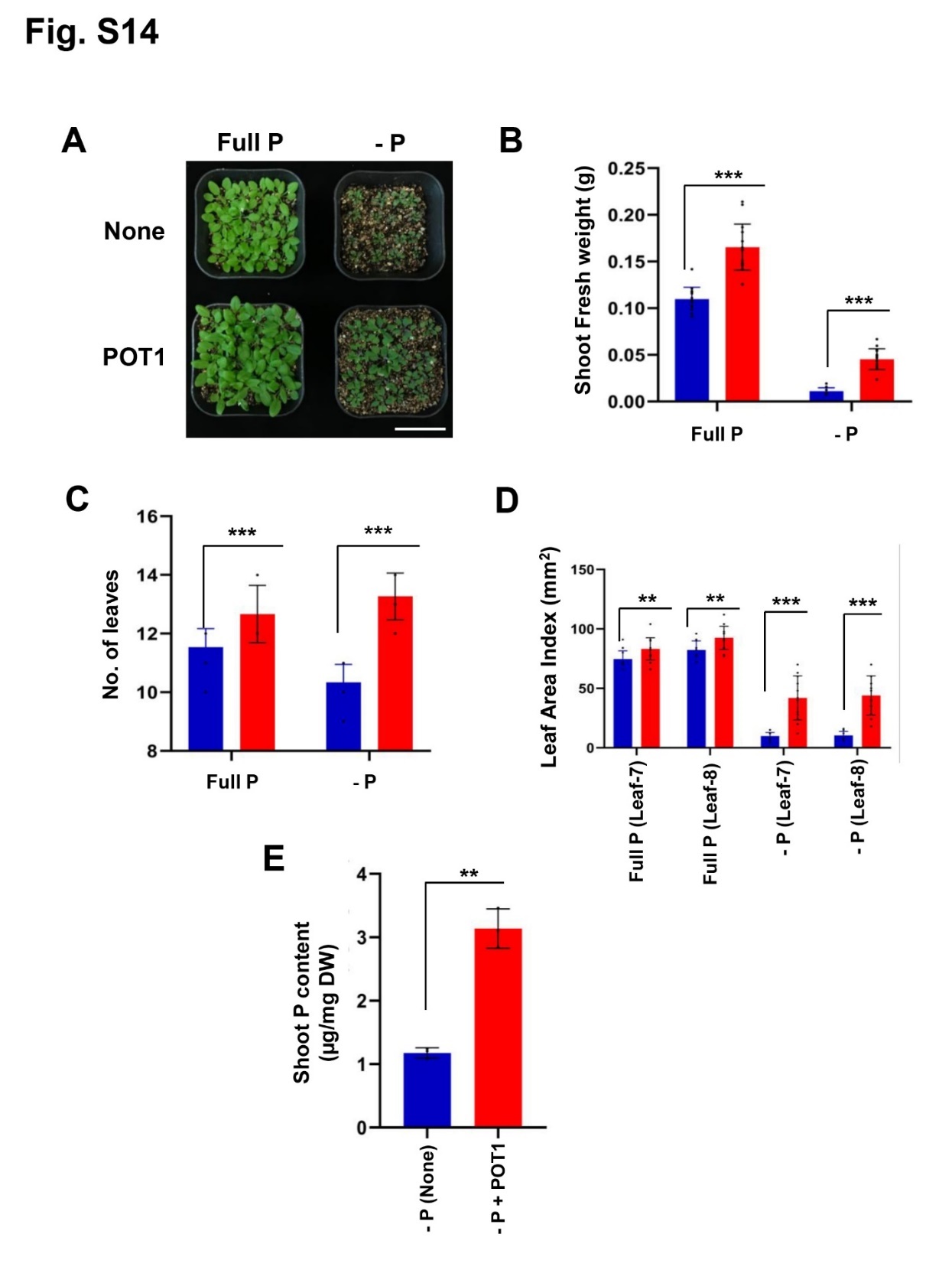


**Supplementary Figure S12.** *P. olsonii* TLL1 (POT1) promotes growth index of Arabidopsis when grown in vermiculite soil supplied with any soluble phosphate sources. **(A)** A representative image of Arabidopsis WT plants grown in full P and no P conditions with and without POT1. Ten-day-old plants were transferred to vermiculite pre-inoculated with POT1 for 14 days. Scale bar represents 1 cm. The bar diagrams represent **(B)** shoot fresh weight, **(C)** number of leaves, and **(D)** leaf area indices of seventh and eighth leaves of Arabidopsis WT plants grown in full P and no P conditions with and without POT1 in vermiculite. The combined data from fifteen independent experiments is shown (n=15; unpaired two-tailed t-test, ****P* <0.001; ***P* <0.01; **P* <0.05). Scale bar represents 5 cm. **(E)** The concentration of total phosphorus content in Arabidopsis shoots grown in full P and no P conditions in vermiculite with and without POT1 (n=3; unpaired two-tailed t-test, ***P* <0.01).


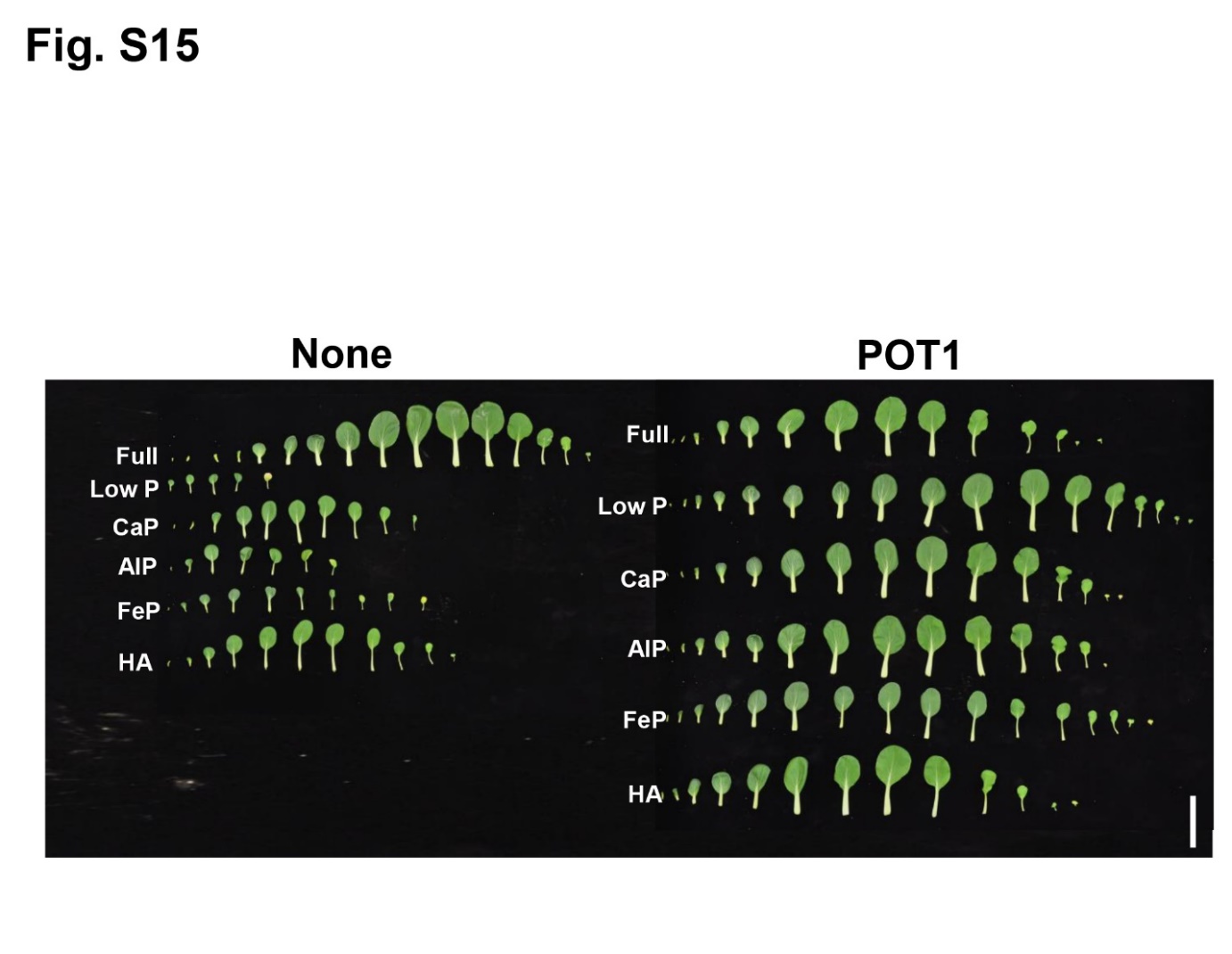


**Supplementary Figure S13.** *P. olsonii* TLL1 (POT1) improves leaf number in Bok Choy plants in vermiculite soil test supplied with P-sufficient and P-limiting conditions, Related to Figure 6. A representative image of leaves of Bok Choy grown in full P, low P, CaP, AlP, FeP and HA as sole source of P with and without POT1. Ten-day-old Bok Choy plants were grown in vermiculite pre-inoculated with POT1 for four weeks. Scale bar represents 1 cm.


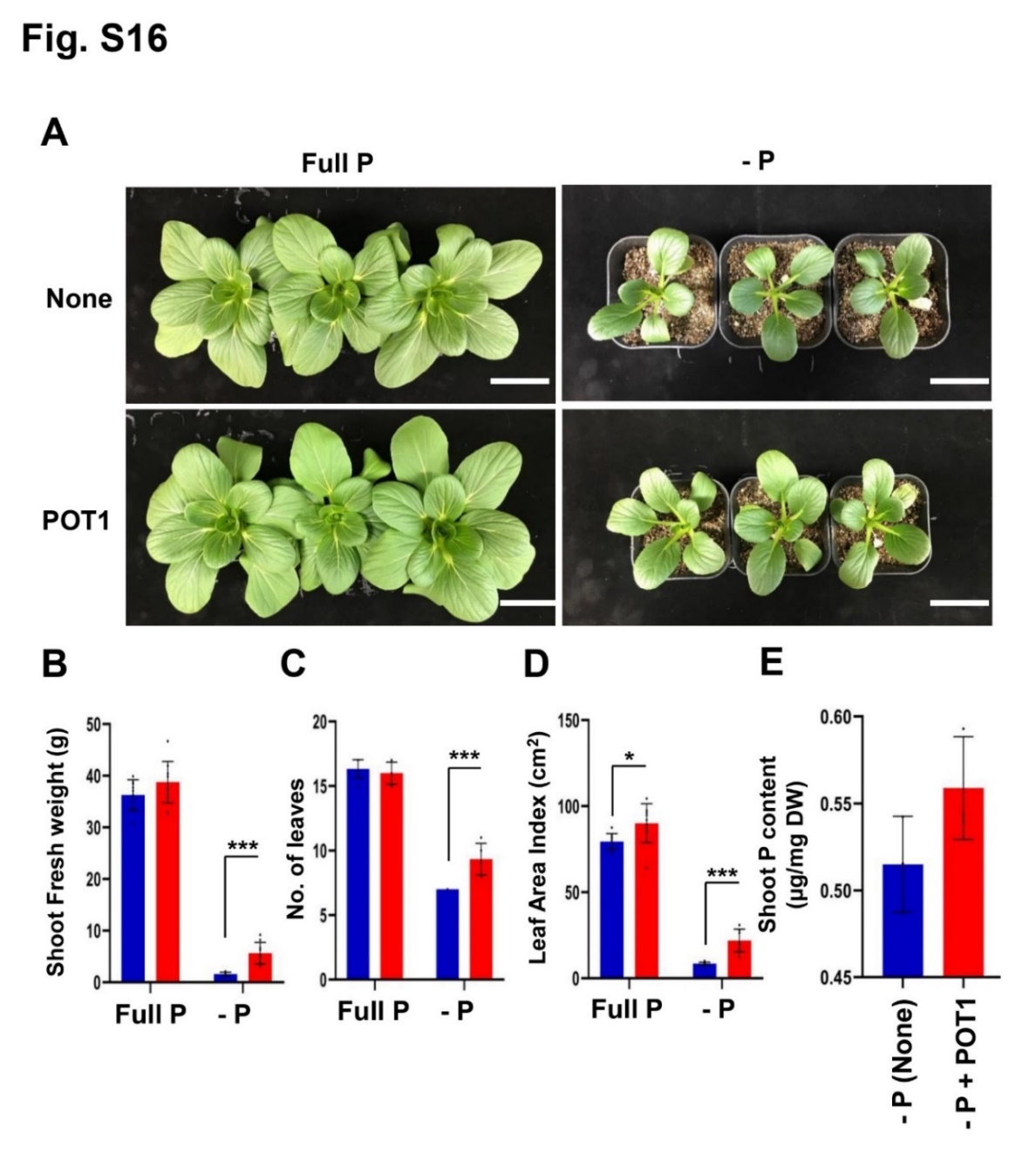


**Supplementary Figure S14.** *P. olsonii* TLL1 (POT1) promotes growth index of leafy vegetable when grown in vermiculite supplied with any soluble phosphate. **(A)** A representative image of Bok Choy grown in full P and no P conditions with and without POT1. Ten-day-old plants were transferred to vermiculite pre-inoculated with and without POT1 for seven days. Scale bar represents 5 cm. The bar diagrams represent **(B)** shoot fresh weight, **(C)** number of leaves, and **(D)** leaf area index of 4^th^ leaf of Bok Choy plants grown in full P and no P conditions with and without POT1 in vermiculite. The combined data from nine independent experiments was analyzed (n=9; unpaired two-tailed t-test, ****P* <0.001; ***P* <0.01; **P* <0.05). **(E)** The concentration of total phosphorus content in Arabidopsis shoots in the presence of POT1 without any phosphate source. Uninoculated plants served as control.


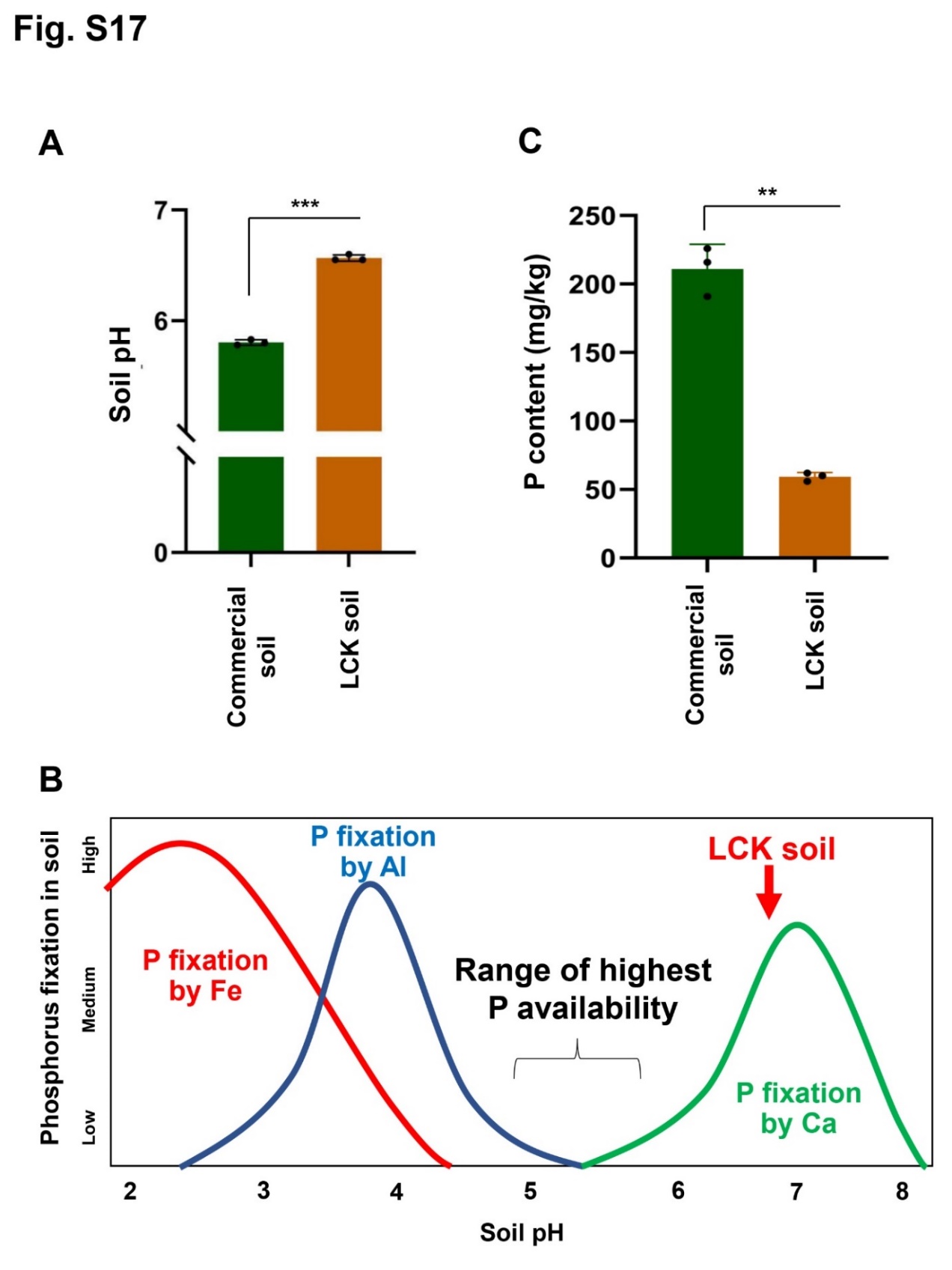


**Supplementary Figure S15.** pH and nutrient analysis of soil used for plant growth experiments. **(A)** Comparison of pH of commercial and Lim Chu Kang (LCK) soil used for plant growth promotion experiments using POT1, **(B)** A representative image of phosphate fixation at different soil pH levels, and **(C)** A bar diagram showing comparison of phosphorus content in commercial as well as Lim Chu Kang soil.


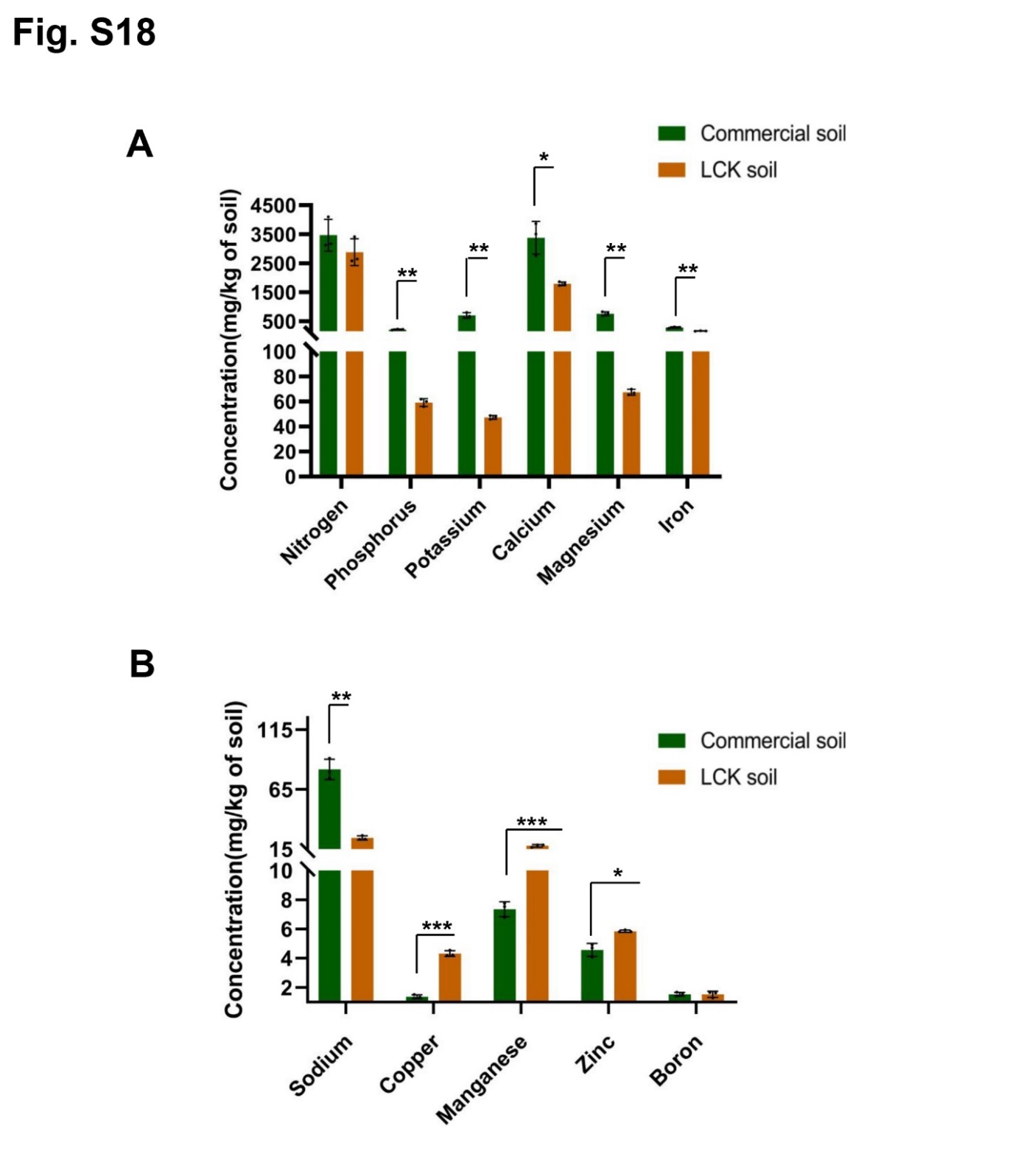


**Supplementary Figure S16.** Soil nutrient profiling of commercial as well as Singapore local soil. A bar diagram showing comparison of nutrient content **(A)** N, P, K, Ca, Mg, Fe and, **(B)** Na, Cu, Mn, Zn and B in commercial as well as Lim Chu Kang soil from three independent experiments (n=3; unpaired two-tailed t-test, ****P* <0.001; ***P* <0.01; **P* <0.05).


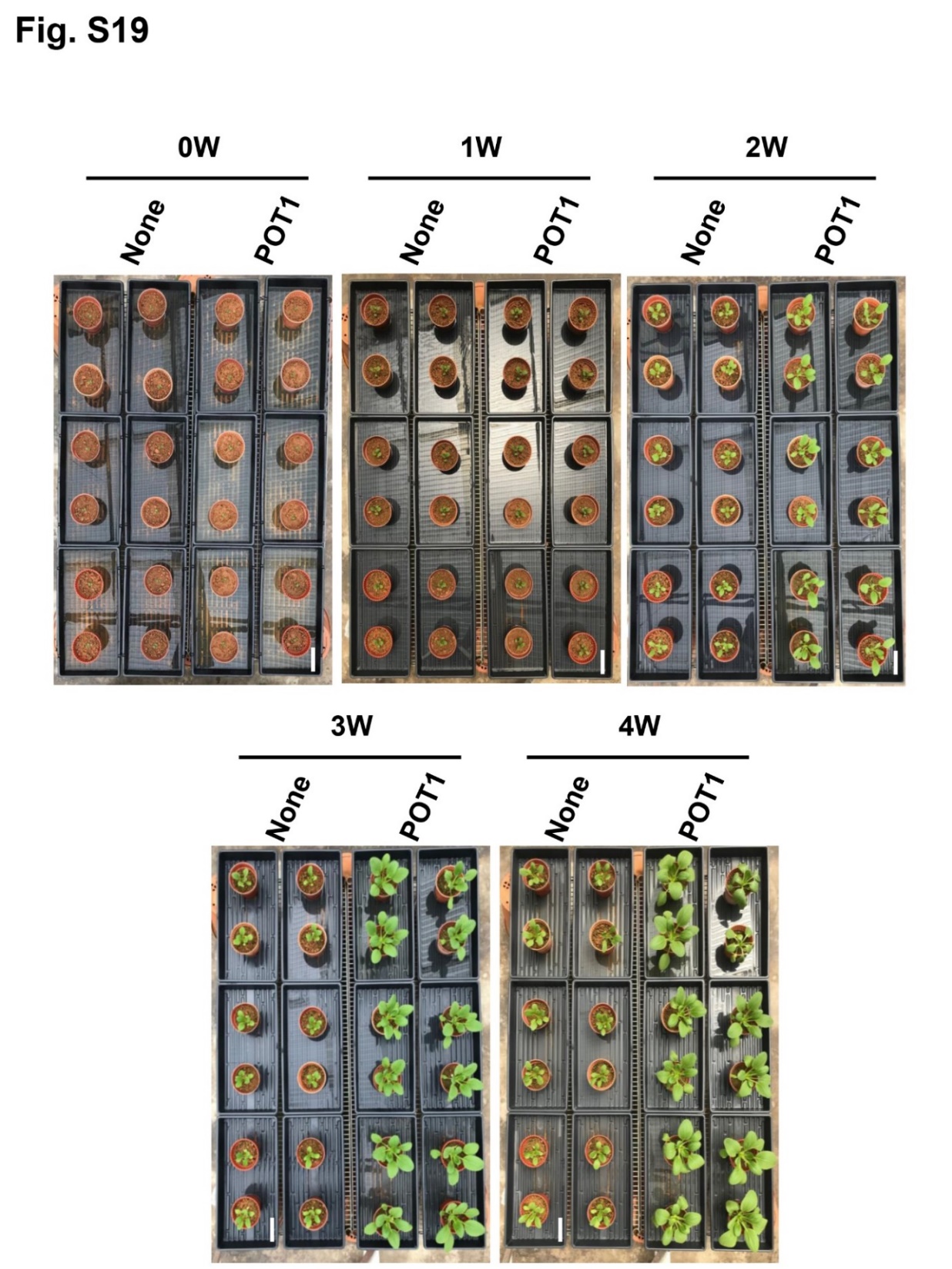


**Supplementary Figure S17.** Growth promotion test of leafy vegetables in Singapore local soil. A representative image of Bok Choy plants grown in Lim Chu Kang soil for four weeks with and without *P. olsonii* TLL1 (POT1).


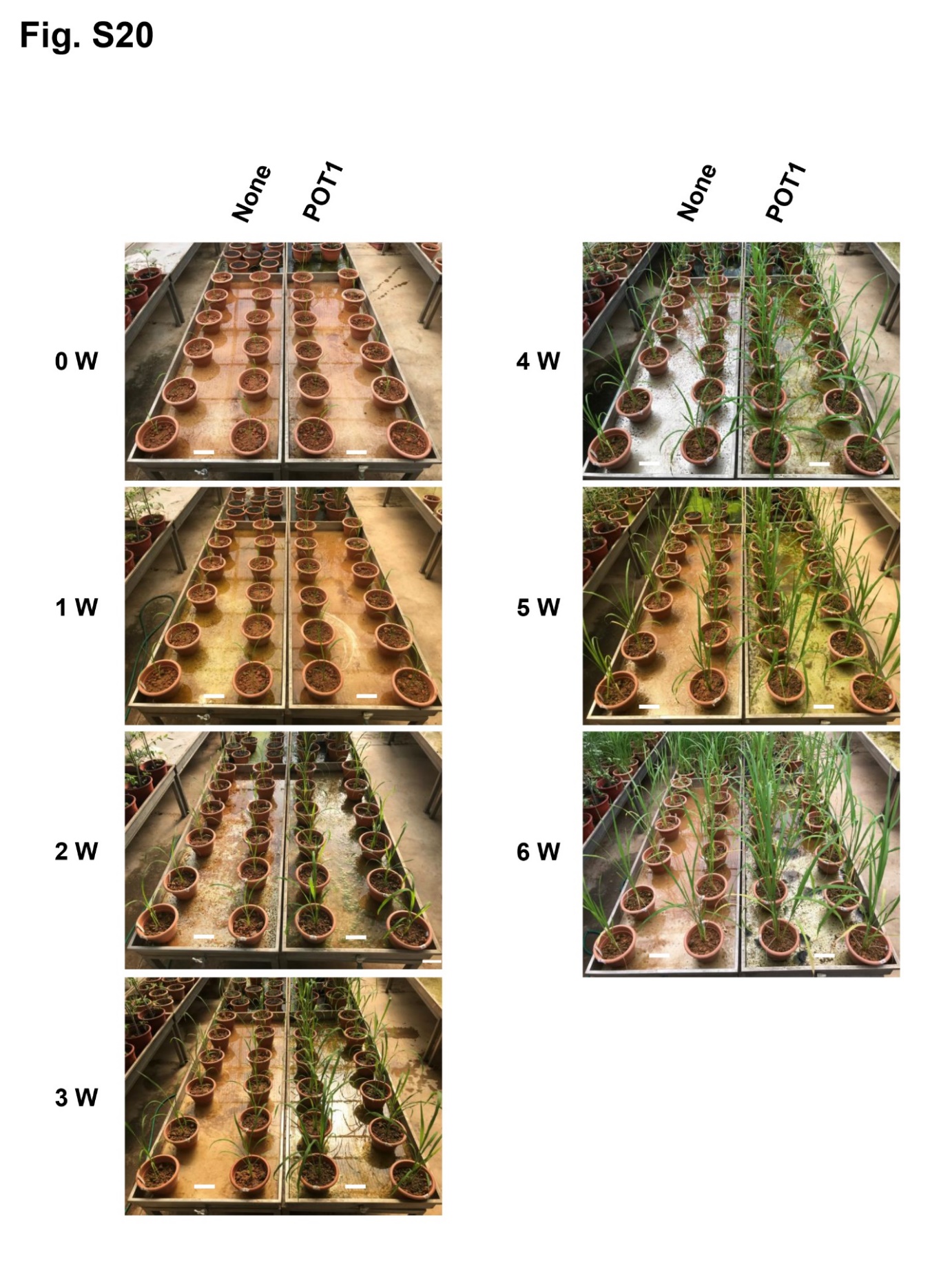


**Supplementary Figure S18.** Growth promotion test of Rice in Singapore local soil. A representative image of Temasek Rice grown in Lim Chu Kang soil for six weeks with and without *P. olsonii* TLL1 (POT1).


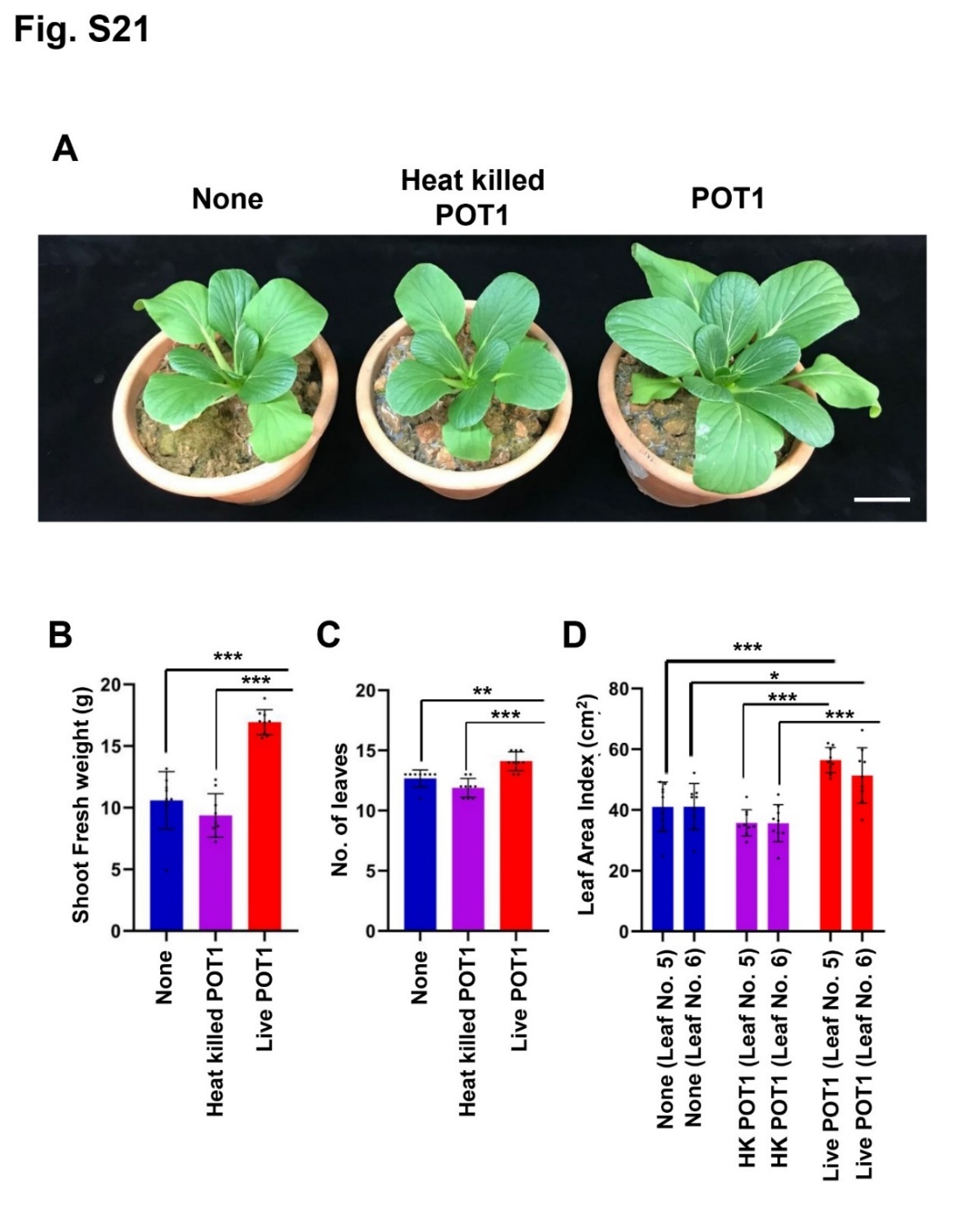


**Supplementary Figure S19.** *P. olsonii* TLL1 (POT1) promotes growth of leafy vegetables in Singapore local soil. A representative image of Bok Choy plants grown in Lim Chu Kang soil with Live POT1, Heat killed POT1 and without POT1. **(A)** Ten-day-old Bok Choy plants were grown in vermiculite pre-inoculated with POT1 for four weeks. The bar diagrams show **(B)** shoot fresh weight, **(C)** number of leaves, and **(D)** leaf area indices of 5^th^ and 6^th^ leaves of Bok Choy plants co-cultivated with POT1 after four weeks. The combined data from nine independent experiments is shown (n=9; unpaired two-tailed t-test, ****P* <0.001; ***P* <0.01; **P* <0.05). Scale bar represents 5 cm.


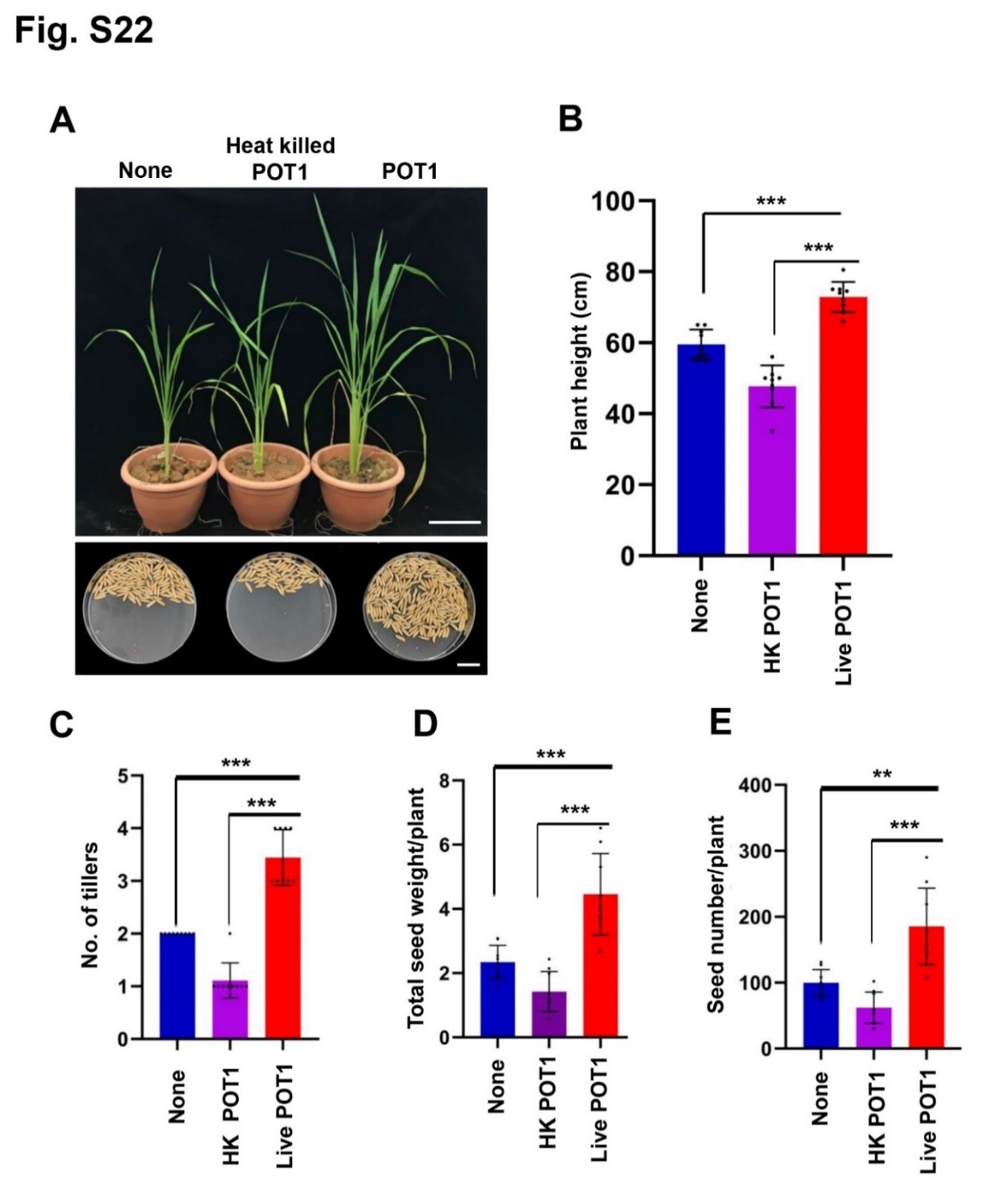


**Supplementary Figure S20.** *P. olsonii* TLL1 (POT1) promotes growth of Rice in Singapore local soil. A representative image of Rice grown in Lim Chu Kang soil with Live POT1, Heat killed POT1 and without POT1. **(A)** Two-week-old Temasek Rice seedlings were grown in LCK soil for three months. The bar diagrams show **(B)** plant height, **(C)** number of tillers after six weeks, **(D)** total seed weight per plant, and **(E)** seed number per plant of Rice co-cultivated with live POT1, heat killed POT1 and without POT1. The combined data from nine independent experiments was analyzed (n=9; unpaired two-tailed t-test, ****P* <0.001; ***P* <0.01; **P* <0.05). Scale bar represents 5 cm.


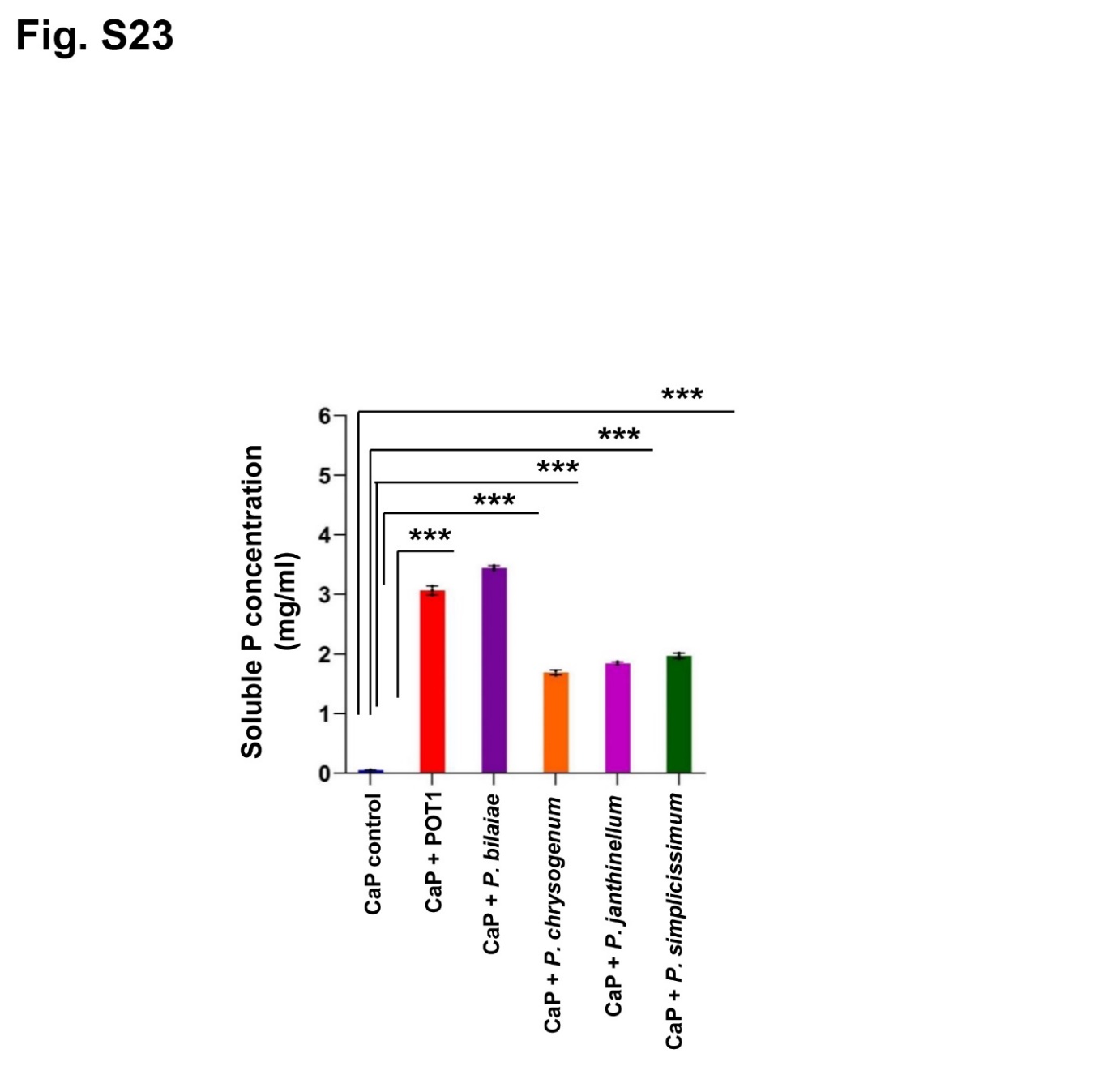


**Supplementary Figure S21.** Comparison of insoluble P solubilization activity of *P. olsonii* TLL1 (POT1) with other *Penicillium* strains. A bar diagram comparing soluble P concentration produced by POT1, *P. bilaiae, P. chrysogenum, P. janthinellum* and *P. simplicissimum* in modified Pikovskaya broth amended with CaP. The error bars show standard deviation. Every treatment included three biological replicates, each of which contained three technical replicates (n=3: unpaired two-tailed t-test, ****P* <0.001; ***P* <0.01; **P* <0.05).


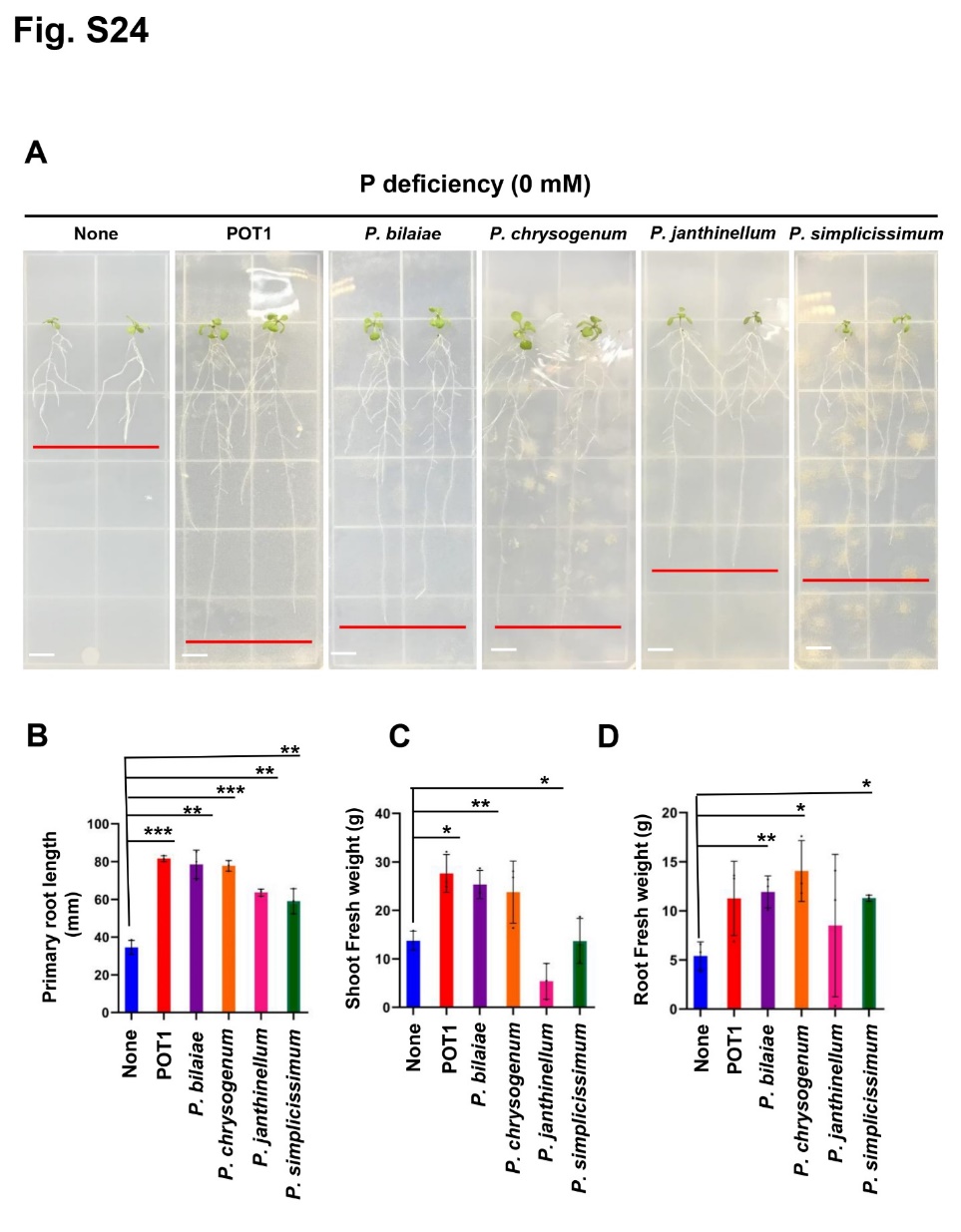


**Supplementary Figure S22.** Comparison of growth promotion activity of *P. olsonii* TLL1 (POT1) with other *Penicillium* strains under P-limiting conditions. **(A)** Arabidopsis plants grown in No P conditions with POT1 and other *Penicillium* strains. Four-day-old plants were transferred to No P media pre-cultured with and without POT1, *P. bilaiae, P. chrysogenum, P. janthinellum* and *P. simplicissimum* under *in vitro* conditions for 10 days. Scale bar represents 1 cm. The bar diagrams show **(B)** primary root length, **(C)** shoot fresh weight, and **(D)** root fresh weight of Arabidopsis WT plants co-cultivated with *P. bilaiae, P. chrysogenum, P. janthinellum* and *P. simplicissimum* in -P conditions for 10 days. The combined data from three independent experiments is shown (n=3; unpaired two-tailed t-test, ****P* <0.001; ***P* <0.01; **P* <0.05).


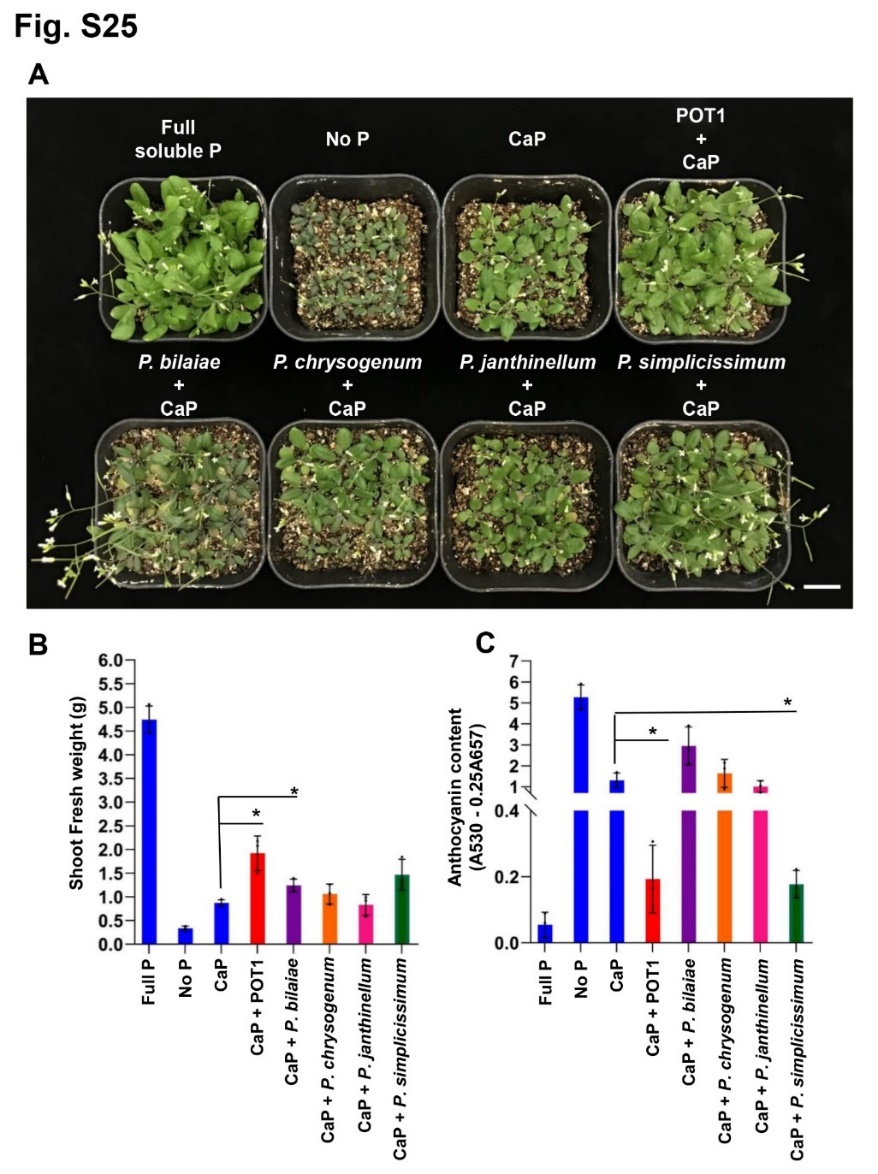


**Supplementary Figure S23.** Comparison of growth promotion activity of *P. olsonii* TLL1 (POT1) with other *Penicillium* strains under insoluble phosphate conditions. **(A)** Arabidopsis plants grown in Full P, No P and CaP conditions with POT1 and other *Penicillium* strains such as *P. bilaiae, P. chrysogenum, P. janthinellum* and *P. simplicissimum*. Ten-day-old plants were transferred to nutrient-poor vermiculite soil pre-inoculated with and without POT1, *P. bilaiae, P. chrysogenum, P. janthinellum* and *P. simplicissimum* under Full P, No P and CaP and were harvested after four weeks. Scale bar represents 1 cm. The bar diagrams show **(B)** shoot fresh weight, and **(C)** anthocyanin content of Arabidopsis WT plants co-cultivated with POT1, *P. bilaiae, P. chrysogenum, P. janthinellum* and *P. simplicissimum* in full P, No P and CaP conditions for 10 days. The combined data from three independent experiments is represented (n=3; unpaired two-tailed t-test, ****P* <0.001; ***P* <0.01; **P* <0.05).

**Supplementary Tables**

**Supplementary Table S1.** Details of primers used for fungus genotyping.

| Locus | Primer ID | Oligonucleotides (5' - 3') | Annealing temp. (⁰C) | Reference |
| --- | --- | --- | --- | --- |
| Internal transcribed spacer region of rDNA (*ITS*) | ITS1 | TCCGTAGGTGA CCTGCGG | 55 | White et al., 1990 |
|  | ITS4 | TCCTCCGCTTATTGATATGC |  |  |
| Translation Elongation Factor 1-alpha (*TEF1α*) | EF1–1018F | GAYTTCATCAAGAACATGAT | 57 | Stielow et al., 2015 |
|  | EF1–1620R | GACGTTGAADCCRACRTTGTC |  |  |
| Small subunit rDNA (*SSU*) | NS1 | GTAGTCATATGCTTGTCTC | 55 | White et al., 1990 |
|  | NS4 | CTTCCGTCAATTCCTTTAAG |  |  |
| Large ribosomal subunit (*LSU*) | LROR-F | ACCCGCTGAACTTAAGC | 58 | Raja et al., 2017 |
|  | LR6R-R | CGCCAGTTCTGCTTACC |  |  |
| Mini chromosome maintenance protein (*MCM7*) | MCM7-709for | ACNMGNGTNTCVGAYGTHAARCC | 55 | Schmitt et al., 2009;  Raja et al., 2011 |
|  | MCM7-1348rev | GATTTDGCIACICCIGGRTCWCCCAT |  |  |

**Supplementary Table S2.** Details of primers used for qPCR analysis of *STOP1, RAE1, ALMT1* and *MATE1*.

|  | Gene |  | Sequence | Reference |
| --- | --- | --- | --- | --- |
| At1G34370 | *AtSTOP1* | F | 5'-TCACATAGCTCTGTTCCAGGGA-3' | Zhang et al., 2019 |
|  |  | R | 5'-ATCAGTCATTCCAGGCTGTGT-3' |  |
|  |  |  |  |  |
| At1G80670 | *AtRAE1* | F | 5′-CCTGATGGTTTGAAGGCGAT-3' |  |
|  |  | R | 5′-CGAATTGGCGATTTGGGTGA-3' |  |
|  |  |  |  |  |
| At1G08430 | *AtALMT1* | F | 5′-CTAGCAAGATTGTCGGGTGC-3′ |  |
|  |  | R | 5′-ACAACGATATCAGCGCGAAC-3' |  |
|  |  |  |  |  |
| At1G51340 | *AtMATE1* | F | 5′-GTAGCTGGCCAGGCAATACTAGC-3' |  |
|  |  | R | 5′-GCCACAAACGGAAGTCCTATGC-3’ |  |

**Supplementary Table S3.** LC-Orbitrap parameters optimized for the parallel reaction monitoring (PRM) mode.

| Compound | Molecular Weight | PRM ion |
| --- | --- | --- |
| Malic acid | 134.09 | 133 🡪 115 |
| Citric acid | 192.12 | 191 🡪 87 |
| Gluconic acid | 196 | 195 🡪 129 |
| Succinic acid | 118.09 | 117 🡪 73 |

**Supplementary Table S4.** Growth index of parameters of Arabidopsis plants when inoculated and uninoculated with *P. olsonii* TLL1 (POT1) under P-sufficient and P-limiting conditions.

|  | Full P | Full P + POT1 | Low P | Low P + POT1 |
| --- | --- | --- | --- | --- |
| Primary root length (mm) | 57.54 | 77.89 | 34.89 | 76.21 |
| *P*-value | 0.003 | | 0.0144 | |
| Shoot weight (mg) | 6.048 | 6.77 | 3.592 | 6.075 |
| *P*-value | 0.3955 | | 0.0059 | |
| Root weight (mg) | 1.617 | 1.653 | 1.068 | 1.986 |
| *P*-value | 0.525 | | 0.021 | |
| Shoot P content (µg/mg DW) | 10.939 | 10.895 | 3.133 | 4.919 |
| *P*-value | 0.916 | | 0.157 | |

**Supplementary Table S5.** Growth index parameters of Arabidopsis plants grown *in vitro* under -P conditions when inoculated and uninoculated with *P. olsonii* TLL1 (POT1).

|  | No P | No P + POT1 |
| --- | --- | --- |
| Primary root length (mm) | 42.19 | 82.90 |
| *P*-value | 0.0005 | |
| Shoot weight (mg) | 3.41 | 3.66 |
| *P*-value | 0.46 | |
| Root weight (mg) | 1.5 | 2.903 |
| *P*-value | 0.012 | |
| Shoot P content (µg/mg DW) | 2.974 | 4.486 |
| *P*-value | 0.023 | |

**Supplementary Table S6.** Gene expression analysis of *RAE1, STOP1, ALMT1 and MATE1* in roots and shoots of Arabidopsis when treated with and without *P. olsonii* TLL1 (POT1) under full P and low P conditions.

|  | *P*-value | | | |
| --- | --- | --- | --- | --- |
|  | *RAE1* | *STOP1* | *ALMT1* | *MATE1* |
| Shoots |  |  |  |  |
| Full P vs Full P + POT1 | 0.3543 | 0.2022 | 0.0101 | 0.7322 |
| Low P vs Low P + POT1 | 0.0712 | 0.1089 | 0.8401 | 0.5801 |
|  |  |  |  |  |
| Roots |  |  |  |  |
| Full P vs Full P + POT1 | 0.1834 | 0.1076 | 0.0401 | 0.0291 |
| Low P vs Low P + POT1 | 0.1186 | 0.5372 | 0.2591 | 0.2144 |

**Supplementary Table S7.** Phosphate solubility index of *P. olsonii* TLL1 (POT1) in Pikovskaya agar and NBRIP media amended with CaP.

|  | PVK | NBRIP |
| --- | --- | --- |
| Phosphate solubility Index | 1.161 | 1.212 |
| *P*-value | 0.172 | |

**Supplementary Table S8.** Soluble P concentration produced by *P. olsonii* TLL1 (POT1) in different inorganic insoluble phosphates such as CaP, AlP, FeP and HA.

|  | CaP  (pH 7.0) | CaP + POT1 (pH 7.0) | AlP  (pH 4.5) | AlP + POT1 (pH 4.5) | FeP  (pH 2.5) | FeP + POT1 (pH 2.5) | HA  (pH 7.0) | HA + POT1 (pH 7.0) |
| --- | --- | --- | --- | --- | --- | --- | --- | --- |
| Soluble P (mg/ml) | 0.0655 | 2.9535 | 0.0993 | 0.9244 | 0.2035 | 0.4355 | -0.1224 | 1.5738 |
| SD | 0.0019 | 0.3449 | 0.0005 | 0.0310 | 0.00516 | 0.00995 | 0.0002 | 0.0455 |
| *P*-value | 0.0047 | | 0.0005 | | 0.00005 | | 0.00024 | |
| Final pH | 6.40 | 5.57 | 4.31 | 2.54 | 2.31 | 1.98 | 6.70 | 5.56 |

**Supplementary Table S9.** Growth index parameters of Arabidopsis in vermiculite soil with insoluble phosphate and phosphate limiting conditions when inoculated and uninoculated with *P. olsonii* TLL1 (POT1).

|  | Shoot weight  (g) | *P*-value | Leaf Area Index-7^th^ leaf (cm^2^) | *P*-value | Leaf Area Index-8^th^ leaf (cm^2^) | *P*-value | Leaf number | *P*-value | Shoot P (µg/mg DW) | *P*-value |
| --- | --- | --- | --- | --- | --- | --- | --- | --- | --- | --- |
| Full P  (0.5 mM) | 0.134 |  | 99.4 |  | 109.533 |  | 11.533 |  | 6.886 |  |
| Full P  (0.5 mM) + POT1 | 0.150 | 0.335 | 66.467 | 0.003 | 68.533 | 0.033 | 12.4 | 0.004 | 1.432 | 0.320 |
| Low P  (0.01 mM) | 0.043 |  | 16.667 |  | 19.4 |  | 9.333 |  | 2.205 |  |
| Low P  (0.01 mM) + POT1 | 0.113 | 0.000 | 44.683 | 0.000 | 46.8 | 0.000 | 11.267 | 0.000 | 4.940 | 0.001 |
| CaP  (0.5 mM) | 0.040 |  | 19.467 |  | 18 |  | 8.533 |  | 2.205 |  |
| CaP  (0.5 mM) + POT1 | 0.114 | 0.000 | 51.067 | 0.000 | 62.933 | 0.000 | 12 | 0.000 | 5.671 | 0.001 |
| AlP  (0.5 mM) | 0.056 |  | 23.583 |  | 24.533 |  | 8.667 |  | 1.465 |  |
| AlP  (0.5 mM) + POT1 | 0.113 | 0.000 | 43.317 | 0.000 | 44.683 | 0.000 | 11.533 | 0.000 | 6.734 | 0.011 |
| FeP  (0.5  mM) | 0.053 |  | 24.3 |  | 22.383 |  | 8.667 |  | 1.554 |  |
| FeP  (0.5 mM) + POT1 | 0.092 | 0.000 | 40.533 | 0.000 | 41.667 | 0.000 | 11.6 | 0.000 | 5.230 | 0.004 |
| HA  (0.5 mM) | 0.044 |  | 26.367 |  | 26.433 |  | 8.667 |  | 1.575 |  |
| HA  (0.5 mM) + POT1 | 0.112 | 0.000 | 45.383 | 0.000 | 44.333 | 0.000 | 11.867 | 0.000 | 5.291 | 0.000 |

**Supplementary Table S10.** Anthocyanin content in shoots of Arabidopsis in vermiculite soil with insoluble phosphate and phosphate limiting conditions when inoculated and uninoculated with *P. olsonii* TLL1 (POT1).

|  | Anthocyanin content (A530-0.25A657) | | | | | |
| --- | --- | --- | --- | --- | --- | --- |
|  | Leaves  (1-4) | *P*-value | Leaves  (5-8) | *P*-value | Leaves (9-12) | *P*-value |
| Full P (0.5 mM) | 0.6377 | 0.0015 | 0.6539 | 0.0092 | 0.7694 | 0.010 |
| Full P (0.5 mM) + POT1 | 0.0640 |  | 0.0626 |  | 0.0747 |  |
| Low P (0.01 mM) | 2.7548 | 0.1134 | 3.1833 | 0.0511 | 0.7942 | 0.0021 |
| Low P (0.01 mM) + POT1 | 0.2743 |  | 0.2051 |  | 0.0332 |  |
| No P (0 mM) | 5.6648 | 0.0317 | 4.3592 | 0.0155 | 1.6779 | 0.0064 |
| No P (0 mM) + POT1 | 0.3665 |  | 0.2914 |  | 0.2891 |  |
| CaP (0.5 mM) | 3.3086 | 0.0285 | 0.8567 | 0.0032 | 1.7327 | 0.0084 |
| CaP (0.5 mM) + POT1 | 0.1968 |  | 0.1704 |  | 0.2557 |  |
| AlP (0.5 mM) | 4.7688 | 0.0283 | 2.6559 | 0.0501 | 2.8603 | 0.0445 |
| AlP (0.5 mM) + POT1 | 0.3460 |  | 0.2452 |  | 0.0655 |  |
| FeP (0.5 mM) | 7.0990 | 0.0081 | 4.9999 | 0.0077 | 2.0267 | 0.0222 |
| FeP (0.5 mM) + POT1 | 0.4527 |  | 0.3195 |  | 0.2461 |  |
| HA (0.5 mM) | 6.6535 | 0.0230 | 2.7213 | 0.0354 | 3.4133 | 0.0000 |
| HA (0.5 mM) + POT1 | 0.6383 |  | 0.3362 |  | 0.2924 |  |

**Supplementary Table S11.** Growth index parameters of Arabidopsis plants grown vermiculite soil under -P conditions when inoculated and uninoculated with *P. olsonii* TLL1 (POT1).

|  | Shoot weight  (g) | *P*-value | Leaf Area Index-7^th^ leaf (cm^2^) | *P*-value | Leaf Area Index-8^th^ leaf (cm^2^) | *P*-value | Shoot P (ug/mg DW) | *P*-value |
| --- | --- | --- | --- | --- | --- | --- | --- | --- |
| Full P (0.5 mM) | 0.107 | 0.000 | 74.733 | 0.008 | 82.367 | 0.004 |  |  |
| Full P (0.5 mM) + POT1 | 0.150 |  | 83.233 |  | 83.233 |  |  |  |
| No P (0 mM) | 0.011 | 0.000 | 9.933 | 0.000 | 10.467 | 0.000 | 1.176 | 0.006 |
| No P (0 mM)  + POT1 | 0.045 |  | 41.933 |  | 44.0 |  | 3.137 |  |

**Supplementary Table S12.** Growth index parameters of Bok Choy in vermiculite soil with insoluble phosphate and phosphate limiting conditions when inoculated and uninoculated with *P. olsonii* TLL1 (POT1).

|  | Shoot weight (g) | *P*-value | Leaf Area Index (cm^2^) | *P*-value | No. of leaves | *P*-value | Shoot P (µg/mg DW) | *P*-value |
| --- | --- | --- | --- | --- | --- | --- | --- | --- |
| Full P  (0.5 mM) | 14.43 | 0.155 | 49.80 | 0.643 | 12.78 | 0.579 | 106 | 0.200 |
| Full P  (0.5 mM) + POT1 | 11.86 |  | 47.264 |  | 12.22 |  | 98.67 |  |
| Low P  (0.01 mM) | 0.58 | 0.000 | 4.4 | 0.000 | 6 | 0.000 | 9.33 | 0.000 |
| Low P  (0.01 mM) + POT1 | 15.22 |  | 52.178 |  | 13.11 |  | 43 |  |
| CaP  (0.5 mM) | 4.98 | 0.000 | 25.988 | 0.000 | 9.33 | 0.000 | 21 | 0.000 |
| CaP  (0.5 mM) + POT1 | 14.99 |  | 54.414 |  | 12.556 |  | 42.33 |  |
| AlP  (0.5 mM) | 1.11 | 0.000 | 7.142 | 0.000 | 6.556 | 0.000 | 9.33 | 0.015 |
| AlP  (0.5 mM) + POT1 | 15.90 |  | 52.638 |  | 13.667 |  | 71.67 |  |
| FeP  (0.5 mM) | 1.80 | 0.000 | 8.01 | 0.001 | 6.667 | 0.000 | 20.33 | 0.122 |
| FeP  (0.5 mM) + POT1 | 6.24 |  | 24.158 |  | 10.222 |  | 37.67 |  |
| HA  (0.5 mM) | 5.23 | 0.000 | 29.318 | 0.000 | 8.889 | 0.000 | 26.67 | 0.038 |
| HA  (0.5 mM) + POT1 | 11.71 |  | 47.969 |  | 12.333 |  | 72 |  |

**Supplementary Table S13.** Growth index parameters of Bok Choy plants grown vermiculite soil under -P conditions when inoculated and uninoculated with *P. olsonii* TLL1 (POT1).

|  | Shoot weight  (g) | *P*-value | No. of leaves | *P*-value | Leaf Area Index (cm^2^) | *P*-value | Shoot P (ug/mg DW) | *P*-value |
| --- | --- | --- | --- | --- | --- | --- | --- | --- |
| Full P (0.5 mM) | 36.25 | 0.153 | 16.33 | 0.3849 | 79.472 | 0.025 |  |  |
| Full P (0.5 mM) + POT1 | 38.76 |  | 16 |  | 90.092 |  |  |  |
| No P (0 mM) | 1.603 | 0.0003 | 7 | 0.0005 | 8.569 | 0.0002 | 0.515 | 0.1334 |
| No P (0 mM)  + POT1 | 5.661 |  | 9.33 |  | 21.973 |  | 0.559 |  |

**Supplementary Table S14.** Soil nutrient profiling of commercial as well as Singapore local soil.

| Nutrients | Concentration (mg/kg of the soil) | | *P*-value |
| --- | --- | --- | --- |
|  | Commercial soil | LCK soil |  |
| Nitrogen | 3465.67 | 2879.33 | 0.23119 |
| Phosphorus | 211 | 59.267 | 0.00381 |
| Potassium | 695.33 | 47.367 | 0.00745 |
| Calcium | 3378 | 1790.333 | 0.03824 |
| Magnesium | 754 | 67.5 | 0.00347 |
| Sodium | 81.7 | 24.4 | 0.00576 |
| Copper | 1.37 | 4.313 | 0.00009 |
| Manganese | 7.357 | 17.8 | 0.00104 |
| Iron | 281 | 163.33 | 0.00713 |
| Zinc | 4.567 | 5.847 | 0.03472 |
| Boron | 1.533 | 1.527 | 0.96483 |

**Supplementary Table S15.** Growth index parameters of Bok Choy in Lim Chu Kang soil when inoculated and uninoculated with *P. olsonii* TLL1 (POT1).

|  | LCK soil | LCK soil + POT1 |
| --- | --- | --- |
| Shoot weight (g) | 3.186 | 9.388 |
| *P*-value | 0.00003 | |
| Leaf Area Index - 3^rd^ leaf (cm^2^) | 9.3833 | 32.35 |
| *P*-value | 0.0000 | |
| Leaf Area Index - 4^th^ leaf (cm^2^) | 12.1017 | 40.372 |
| *P*-value | 0.0000 | |
| Leaf number | 7.417 | 10.833 |
| *P*-value | 0.0000 | |
| Shoot P content (µg/mg DW) | 15 | 53.333 |
| *P*-value | 0.0001 | |

**Supplementary Table S16.** Growth index parameters of Temasek Rice in Lim Chu Kang soil when inoculated and uninoculated with *P. olsonii* TLL1 (POT1).

|  | LCK soil | LCK soil + POT1 |
| --- | --- | --- |
| Plant height (cm) | 66.842 | 79.725 |
| *P*-value | 0.000 | |
| No. of tillers | 2 | 3.167 |
| *P*-value | 0.000 | |
| Seed weight (g) | 2.286 | 3.98 |
| *P*-value | 0.004 | |
| No. of seeds | 103 | 184 |
| *P*-value | 0.001 | |
| Shoot P content  (µg/mg dry weight) | 12 | 16.33 |
| *P*-value | 0.039 | |

**Supplementary Table S17.** Growth index parameters of Bok Choy in Lim Chu Kang soil when inoculated with live *P. olsonii* TLL1 (POT1), heat-killed POT1 and uninoculated with POT1.

|  | LCK soil | LCK soil +  HK POT1 | LCK soil +  Live POT1 |
| --- | --- | --- | --- |
| Shoot weight (g) | 10.60 | 9.37 | 16.95 |
| *P*-value |  | 0.000 | 0.000 |
| Leaf Area Index – 5^th^ leaf (cm^2^) | 41.04 | 35.786 | 56.42 |
| *P*-value |  | 0.000 | 0.000 |
| Leaf Area Index – 6^th^ leaf (cm^2^) | 41.126 | 35.662 | 51.368 |
| *P*-value |  | 0.001 | 0.020 |
| Leaf number | 12.67 | 11.89 | 14.11 |
| *P*-value |  | 0.000 | 0.000 |

**Supplementary TableS18.** Growth index parameters of Temasek Rice in Lim Chu Kang soil when inoculated with live *P. olsonii* TLL1 (POT1), heat-killed POT1 and uninoculated with POT1.

|  | LCK soil | LCK soil +  HK POT1 | LCK soil +  Live POT1 |
| --- | --- | --- | --- |
| Plant height (cm) | 59.5 | 47.72 | 72.89 |
| *P*-value |  | 0.000 | 0.000 |
| No. of tillers | 2 | 1.11 | 3.444 |
| *P*-value |  | 0.000 | 0.000 |
| Seed weight (g) | 2.345 | 1.423 | 4.454 |
| *P*-value |  | 0.001 | 0.000 |
| No. of seeds | 99.778 | 62.111 | 185.56 |
| *P*-value |  | 0.002 | 0.0001 |

**Supplementary Table S19.** Comparison of tricalcium phosphate (CaP) solubilization activity of *P. olsonii* TLL1 (POT1) with other *Penicillium* strains.

|  | Soluble P (mg/ml) | *P*-value |
| --- | --- | --- |
| CaP (None) | 0.05 |  |
| CaP + POT1 | 3.06 | 0.0002 |
| CaP + *P. bilaiae* | 3.44 | 0.0000 |
| CaP + *P. chrysogenum* | 1.69 | 0.0002 |
| CaP + *P. janthinellum* | 1.84 | 0.0001 |
| CaP+ *P. simplicissimum* | 1.97 | 0.0002 |

**Supplementary Table S20.** Comparison of growth index parameters of Arabidopsis plants when inoculated and uninoculated with *P. olsonii* TLL1 (POT1) and other *Penicillium* strains under P-limiting conditions.

|  | No P | No P+  POT1 | No P +  *P. bilaiae* | No P + *P. chrysogenum* | No P + *P. janthinellum* | No P + *P. simplicissimum* |
| --- | --- | --- | --- | --- | --- | --- |
| Primary root length (mm) | 34.51 | 81.61 | 78.55 | 77.78 | 63.63 | 59.06 |
| *P*-value |  | 0.000 | 0.003 | 0.000 | 0.001 | 0.010 |
| Shoot weight (mg) | 13.77 | 27.63 | 25.33 | 23.77 | 5.37 | 13.7 |
| *P*-value |  | 0.012 | 0.007 | 0.104 | 0.039 | 0.983 |
| Root weight (mg) | 5.4 | 11.27 | 11.93 | 14.07 | 8.5 | 11.3 |
| *P*-value |  | 0.101 | 0.007 | 0.025 | 0.539 | 0.016 |

**Supplementary Table S21.** Comparison of growth index parameters of Arabidopsis in vermiculite soil with insoluble tricalcium phosphate and phosphate limiting conditions when inoculated and uninoculated with *P. olsonii* TLL1 (POT1) and other *Penicillium* strains.

|  | Shoot weight  (g) | *P*-value | Anthocyanin content  (A530-0.25A657) | *P*-value |
| --- | --- | --- | --- | --- |
| Full P (0.5 mM) | 4.743 |  | 0.055 |  |
| No P (0 mM) | 0.333 |  | 5.272 |  |
| Ca P (0.5 mM) | 0.87 |  | 1.310 |  |
| CaP (0.5 mM) +  POT1 | 1.92 | 0.034 | 0.193 | 0.022 |
| CaP (0.5 mM) +  *P. bilaiae* | 1.243 | 0.023 | 2.95 | 0.074 |
| CaP (0.5 mM) +  *P. chrysogenum* | 1.06 | 0.255 | 1.638 | 0.503 |
| CaP (0.5 mM) +  *P. janthinelleum* | 0.83 | 0.788 | 1.016 | 0.313 |
| CaP (0.5 mM) +  *P. simplicissimum* | 1.463 | 0.081 | 0.178 | 0.028 |
